# Leveraging multiple transcriptome assembly methods for improved gene structure annotation

**DOI:** 10.1093/gigascience/giy093

**Published:** 2018-07-24

**Authors:** Luca Venturini, Shabhonam Caim, Gemy George Kaithakottil, Daniel Lee Mapleson, David Swarbreck

**Affiliations:** 1 Earlham Institute, Norwich Research Park, NR47UZ, Norwich, United Kingdom; 2 Quadram Institute Biosciences, Norwich Research Park, NR47UA, Norwich, United Kingdom

**Keywords:** RNA-seq, transcriptome, assembly, genome annotation

## Abstract

**Background:**

The performance of RNA sequencing (RNA-seq) aligners and assemblers varies greatly across different organisms and experiments, and often the optimal approach is not known beforehand.

**Results:**

Here, we show that the accuracy of transcript reconstruction can be boosted by combining multiple methods, and we present a novel algorithm to integrate multiple RNA-seq assemblies into a coherent transcript annotation. Our algorithm can remove redundancies and select the best transcript models according to user-specified metrics, while solving common artifacts such as erroneous transcript chimerisms.

**Conclusions:**

We have implemented this method in an open-source Python3 and Cython program, Mikado, available on GitHub.

## Background

The annotation of eukaryotic genomes is typically a complex process that integrates multiple sources of extrinsic evidence to guide gene predictions. Improvements and cost reductions in the field of nucleic acid sequencing now make it feasible to generate a genome assembly and to obtain deep transcriptome data, even for nonmodel organisms. However, for many of these species, there are only minimal expressed sequence tag (EST) and cDNA resources and limited availability of proteins from closely related species. In these cases, transcriptome data from high-throughput RNA sequencing (RNA-seq) provides a vital source of evidence to aid gene structure annotation. A detailed map of the transcriptome can be built from a range of tissues, developmental stages, and conditions, aiding the annotation of transcription start sites, exons, alternative splice variants, and polyadenylation sites.

Currently, one of the most commonly used technologies for RNA-seq is Illumina sequencing, which is characterized by extremely high throughput and relatively short read lengths. Since its introduction, numerous algorithms have been proposed to analyze its output. Many of these tools focus on the problem of assigning reads to known genes to infer their abundance [2–5] or of aligning them to their genomic locus of origin [6–8]. Another challenging task is the reconstruction of the original sequence and genomic structure of transcripts directly from sequencing data. Many approaches developed for this purpose leverage genomic alignments [9–12], although there are alternatives based instead on *de novo* assembly [10, 13, 14]. While these methods focus on how to analyze a single dataset, related research has examined how to integrate assemblies from multiple samples. While some researchers advocate for merging together reads from multiple samples and assembling them jointly [10], others have developed methods to integrate multiple assemblies into a single coherent annotation [9, 15].

The availability of multiple methods has generated interest in understanding the relative merits of each approach [16–18]. The correct reconstruction of transcripts is often hampered by the presence of multiple isoforms at each locus and the extreme variability of expression levels, and therefore in sequencing depth, within and across gene loci. This variability also affects the correct identification of transcription start and end sites, as sequencing depth typically drops near the terminal ends of transcripts. The issue is particularly severe in compact genomes, where genes are clustered within small intergenic distances. Further, the presence of tandemly duplicated genes can lead to alignment artifacts that then result in multiple genes being incorrectly reconstructed as a fused transcript. As observed in a comparison performed by the RGASP consortium [19], the accuracy of each tool depends on how it corrects for each of these potential sources of errors. However, it also depends on other external factors such as the quality of the input sequencing data as well as on species-dependent characteristics, such as intron sizes and the extent of alternative splicing. It has also been observed that no single method consistently delivers the most accurate transcript set when tested across different species. Therefore, none of them can be determined *a priori* as the most appropriate for a given experiment [20]. These considerations are an important concern in the design of genome annotation pipelines, as transcript assemblies are a common component of evidence-guided approaches that integrate data from multiple sources (e.g., cDNAs, protein, or whole genome alignments). The quality and completeness of the assembled transcript set can therefore substantially have an impact on downstream annotation.

Following these studies, various approaches have been proposed to determine the best assembly using multiple measures of assembly quality [20, 21] or to integrate RNA-seq assemblies generated by competing methods [22–24]. In this study, we show that alternative methods not only have different strengths and weaknesses, but that they also often complement each other by correctly reconstructing different subsets of transcripts. Therefore, methods that are not the best overall might nonetheless be capable of outperforming the “best” method for a subset of loci. An annotation project typically integrates datasets from a range of tissues or conditions or may utilize public data generated with different technologies (e.g., Illumina, Pacific Biosciences [PacBio]) or sequencing characteristics (e.g., read length, strand specificity, ribo-depletion). In such cases, it is not uncommon to produce at least one set of transcript assemblies for each of the different sources of data, assemblies that then need to be reconciled. To address these challenges, we developed MIKADO [1], an approach to integrate transcript assemblies. The tool defines loci, scores transcripts, determines a representative transcript for each locus, and finally returns a set of gene models filtered to individual requirements, e.g., removing transcripts that are chimeric, fragmented, or with short or disrupted coding sequences. Our approach was shown to outperform both stand-alone methods and those that combine assemblies, by returning more transcripts reconstructed correctly and less chimeric and unannotated genes.

## Results and Discussion

### Assessment of RNA-seq based transcript reconstruction methods

We evaluated the performance of four commonly utilized transcript assemblers: Cufflinks, StringTie, CLASS2, and Trinity. Their behavior was assessed in four species using as input data RNA-seq reads aligned with two alternative leading aligners, TopHat2 and STAR. We generated 32 different transcript assemblies, 8 per species. In line with the previous RGASP evaluation, we performed our tests on the three metazoan species of *Caenhorabditis elegans*, *Drosophila melanogaster*, and *Homo sapiens* using RNA-seq data from that study as input. We also added to the panel a plant species, *Arabidopsis thaliana*, to assess the performance of these tools on a non-metazoan genome. Each species has undergone extensive manual curation to refine gene structures. Importantly, these annotations exhibit very different gene characteristics in terms of their proportion of single exon genes, average intron lengths, and number of annotated transcripts per gene (Supplementary Table S1). Similar to previous studies [19, 25], we based our initial assessment on real rather than simulated data to ensure we captured the true characteristics of RNA-seq data. Prediction performance was benchmarked against the subset of annotated transcripts, with all exons and introns (minimum 1X coverage) identified by at least one of the two RNA-seq aligners.

The number of transcripts assembled varied substantially across methods, with StringTie and Trinity generally reporting a greater number of transcripts (Supplementary Fig. S1). Assembly with Trinity was performed using the genome-guided *de novo* method where RNA-seq reads are first partitioned into loci ahead of *de novo* assembly. This approach is in contrast to the genome-guided approaches employed by the other assemblers that allow small drops in read coverage to be bridged and enable the exclusion of retained introns and other lowly expressed fragments. As expected, Trinity annotated more fragmented loci with a higher proportion of mono-exonic genes (Supplementary Fig. S1).

The accuracy of transcript reconstruction was measured using recall and precision. For any given feature (nucleotide, exon, transcript, gene), recall is defined as the percentage of correctly predicted features out of all expressed reference features, whereas precision is defined as the percentage of all features that correctly match a feature present in the reference. In line with previous evaluations, we found that accuracy varied considerably among methods, with clear trade-offs between recall and precision (Supplementary Fig. S2). For instance, CLASS2 emerged as the most precise of all methods tested, but its precision came at the cost of reconstructing fewer transcripts overall. In contrast, Trinity and StringTie often outperformed the recall of CLASS2 but were also much more prone to yield transcripts absent from the curated public annotations (Supplementary Figs. S2, S3). Although many of these might be real, yet-unknown transcripts, the high number of chimeric transcripts suggests that these novel models be treated with suspicion. Notably, the performance and the relative ranking of the methods differed among the four species (Table1). We found CLASS2 and StringTie to be overall the most accurate (with either aligner); however, exceptions were evident. For instance, the most accurate method in *D. melanogaster* (CLASS2 in conjunction with Tophat alignments) performed worse than any other tested method in *A. thaliana*. The choice of RNA-seq aligner also substantially impacted assembly accuracy, with clear differences between the two when used in conjunction with the same assembler.

**Table 1:** Cumulative z-score for each method aggregating individual z-scores based on base, exon, intron, intron chain, transcript, and gene F1 score .

	*A. thaliana*		*C. elegans*		*D. melanogaster*		*H. sapiens*		All methods	
Method	Z-score	Rank	Z-score	Rank	Z-score	Rank	Z-score	Rank	Z-score	Rank
CLASS2 (STAR)	**7.627**	**1**	**7.309**	**1**	−3.310	6	**5.258**	**1**	**16.884**	**1**
StringTie (TopHat2)	0.584	4	5.502	3	6.612	2	3.199	3	15.897	2
CLASS2 (TopHat2)	*−5.542*	*8*	6.698	2	**9.314**	**1**	4.998	2	15.738	3
StringTie (STAR)	2.621	3	−2.197	4	1.587	3	2.991	4	5.001	4
Cufflinks (STAR)	2.716	2	−2.306	5	−1.730	5	1.037	5	−0.283	5
Cufflinks (TopHat2)	−0.526	5	*−5.363*	*8*	−1.504	4	−0.993	6	−8.386	6
Trinity (STAR)	−4.120	7	−5.079	7	−4.762	7	−3.417	7	−17.458	7
Trinity (TopHat2)	−3.280	6	−4.833	6	*−6.206*	*8*	*−13.073*	*8*	*−27.392*	*8*

Top ranked method in bold, bottom ranked method underlined and in italics.

Across the four species and depending on the aligner used, 22% to 35% of transcripts could be reconstructed by any combination of aligner and assembler (Supplementary Table S2). However, some genes were recovered only by a subset of the methods (Supplementary Table S2), with on average 5% of the genes being fully reconstructable only by one of the available combinations of aligner and assembler. Closer inspection of the data shows that this effect is not due to a single assembler having greater efficiency. Rather, each tool is shown to be the only one capable of correctly reconstructing hundreds of the expressed transcripts (Supplementary Fig. S4). Taking the union of genes fully reconstructed by any of the methods shows that an additional 14.92% to 19.08% of genes could be recovered by an approach that would integrate the most sensitive assembly with less comprehensive methods. This complementarity manifests as well in relation to genes missed by any particular method. While each approach failed to reconstruct several hundred genes on average, the majority of these models could be fully or partially reconstructed by an alternative method (Supplementary Fig. S3A). Another class of error is artifactual fusion/chimeric transcripts that chain together multiple genes. These artifacts usually arise from an incorrect identification of start and end sites during transcript reconstruction. This is an issue that appears most prominently in compact genomes with smaller intergenic distances [10]. Among the methods tested, Cufflinks was particularly prone to this class of error, while Trinity and CLASS2 assembled far fewer such transcripts. Again, alternative methods complemented each other, with many genes fused by one assembler being reconstructed correctly by another approach (Supplementary Fig. S3B). Finally, the efficiency of transcript reconstruction depends on coverage, a reflection of sequencing depth and expression level. Methods in general agree on the reconstruction of well-expressed genes, while they show greater variability with transcripts that are present at lower expression levels. Even at high expression levels, though, only a minority of genes can be reconstructed correctly by every tested combination of aligner and assembler (Supplementary Fig. S5). Our results underscore the difficulty of transcript assembly and highlight advantageous features of specific methods. A naive combination of the output of all methods would yield the greatest sensitivity, but at the cost of a decrease in precision as noise from erroneous reconstructions accumulates. Indeed, this is what we observe: in all species, while the recall of the naive combination markedly improves, even upon the most sensitive method, the precision decreases (Supplementary Fig. S2). As transcript reconstruction methods exhibit idiosyncratic strengths and weaknesses, an approach that can integrate multiple assemblies can potentially lead to a more accurate and comprehensive set of gene models.

### Overview of the Mikado method

Mikado provides a framework for integrating transcripts from multiple sources into a consolidated set of gene annotations. Our approach assesses, scores (based on user configurable criteria), and selects transcripts from a larger transcript pool, leveraging transcript assemblies generated by alternative methods or from multiple samples and sequencing technologies.

The software takes as input transcript structures in standard formats such as General Transfer Format (GTF) and General Feature Format 3 (GFF3), with optionally Basic Local Alignment Search Tool (BLAST) similarity scores or a set of high-quality splice junctions. Using this information, Mikado will then define gene loci and their associated transcripts. Each locus will be characterized by a primary transcript, i.e., the transcript in the region that best fits the requirements specified by the user and that therefore receives the highest score. If any suitable alternative splicing event for the primary transcript is available, Mikado will add it to the locus. The software is written in python3 and Cython, and extensive documentation is available on Read The Docs [26].

Mikado is composed of three core programs (prepare, serialize, pick) executed in series. The *Mikado prepare* step validates and standardizes transcripts, removing exact duplicates and artifactual assemblies such as those with ambiguous strand orientation (as indicated by canonical splicing). During the *Mikado serialize* step, data from multiple sources are brought together inside a common database. By default, Mikado analyses and integrates three types of data: open reading frames (ORFs) currently identified via TransDecoder, protein similarity derived through BLASTX or Diamond, and high-quality splice junctions identified using tools such as Portcullis [27] or Stampy [28]. The selection phase (Mikado pick) groups transcripts into loci and calculates for each transcript more than 50 numerical and categorical metrics based on either external data (e.g., BLAST support) or intrinsic qualities relating to coding sequence (CDS), exon, intron, or untranslated region (UTR) features (summarized in Supplementary Table S3).

**Figure 1:**
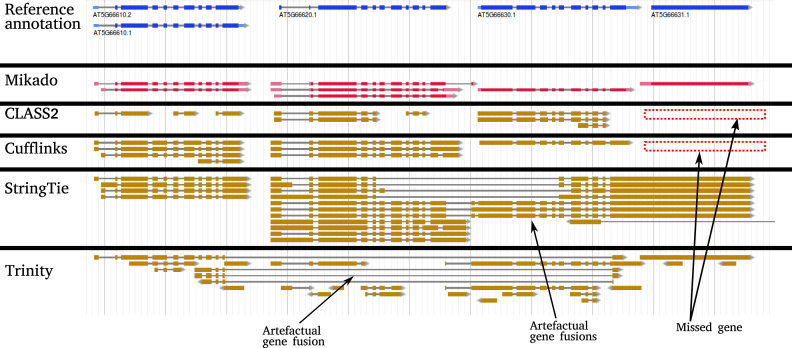
The algorithm employed by Mikado is capable of solving complex loci with multiple potential assemblies. This locus in *A. thaliana* is particularly challenging as an ancestral gene in the locus tandemly duplicated into the current AT5G66610, AT5G66620, and AT5G66630 genes. Due to these difficulties, no single assembler was capable of reconstructing all loci correctly. For instance, Trinity was the only method that correctly assembled AT5G66631, but it failed to reconstruct any other transcript correctly. The reverse was true for Cufflinks, which correctly assembled the three duplicated genes but completely missed the monoexonic AT566631. By choosing between different alternative assemblies, Mikado was capable to provide an evidence-based annotation congruent to the TAIR10 models.

While some metrics are inherent to each transcript (e.g., the cDNA length), others depend on the context of the locus the transcript is placed in. A typical example would be the proportion of introns of the transcript relative to the number of introns associated with the genomic locus. Such values are dependent on the loci grouping and can change throughout the computation as transcripts are moved into a different locus or filtered out. Notably, the presence of ORFs is used in conjunction with protein similarity to identify and resolve fusion transcripts. Transcripts with multiple ORFs are marked as candidate false fusions. Homology to reference proteins is then optionally used to determine whether the ORFs derive from more than one gene. If the fusion event is confirmed, the transcript is split into multiple transcripts (Fig.1).

To determine the primary transcript at a locus, Mikado assigns a score for each metric of each transcript by assessing its value relative to all other transcripts associated with the locus. Once the highest scoring transcript for the group has been selected, Mikado will exclude all transcripts that are directly intersecting it; if any remain, it will iteratively select the next best scoring transcripts, pruning the graph until all nonintersecting transcripts have been selected. This iterative strategy ensures that no locus is excluded if, e.g., there are unresolved read-through events that would connect two or more gene loci. Grouping and filtering happen in multiple sequential phases, each defined by different rules for clustering transcripts into loci (see the Methods section). After the gene loci and associated primary transcripts have been defined, Mikado will look for potential alternative splicing events. Only transcripts that can be unambiguously assigned to a single gene locus will be considered for this phase. Mikado will add to the locus only transcripts whose structures are nonredundant with those already present and that are valid alternative splicing events for the primary transcript, as defined by the class codes [29]. Moreover, Mikado will discard any transcript whose score is too low when compared to the primary (by default, only transcripts with a score of 50% or more of the primary transcript will be considered). The process is controlled by a configuration file that determines desirable gene features, allowing the user to define criteria for transcript filtering and scoring as well as specifying minimum requirements for potential alternative splicing events. The online documentation contains details on the format of the configuration file [30] and provides a tutorial on how to create such files or adapt existing ones to new projects [31].

Candidate isoforms will be ranked according to their score and considered in decreasing order, with a cap on the maximum number of alternative isoforms and on the minimum score for a candidate to be considered valid (by default, at a minimum 50% of the score of the primary transcript). Mikado will add to the locus only transcripts whose structures are nonredundant with those already present and that are valid alternative splicing events for the primary transcript, as defined by class codes [29]. The process is controlled by a configuration file that determines desirable gene features, allowing the user to define criteria for transcript filtering and scoring as well as specifying minimum requirements for potential alternative splicing events.

**Figure 2:**
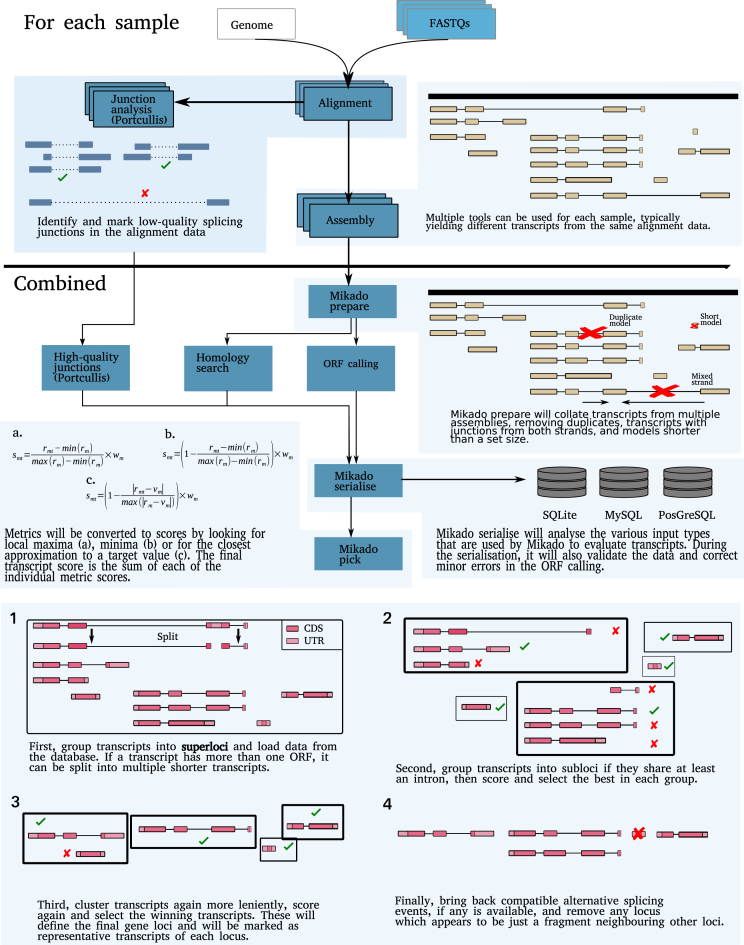
Schematic representation of the Mikado workflow.

We also developed a Snakemake-based pipeline, Daijin, in order to drive Mikado, including the calls to external programs to calculate ORFs and protein homology. Daijin works in two independent stages, assemble and mikado. The former stage enables transcript assemblies to be generated from the read datasets using a choice of read alignment and assembly methods. In parallel, this part of the pipeline will also calculate reliable junctions for each alignment using Portcullis. The latter stage of the pipeline drives the steps necessary to execute Mikado, both in terms of the required steps for our program (prepare, serialize, pick) and of the external programs needed to obtain additional data for the picking stage (currently, homology search and ORF detection). A summary of the Daijin pipeline is provided in Fig. 2.

### Performance of Mikado

To provide a more complete assessment, we evaluated the performance of Mikado on both simulated and real data. While real data represent more fully the true complexity of the transcriptome, simulated data generate a known set of transcripts to enable a precise assessment of prediction quality. For our purposes, we used SPANKI to simulate RNA-seq reads for all four species, closely matching the quality and expression profiles of the corresponding real data. Simulated reads were aligned and assembled following the same protocol that was used for real data, described above. For each of the four species under analysis, we also obtained reference-quality protein sequences from related species to inform the homology search through BLAST; details on our selection can be found in Supplementary Table S4. Mikado was then used to integrate the four different transcript assemblies for each alignment.

**Figure 3:**
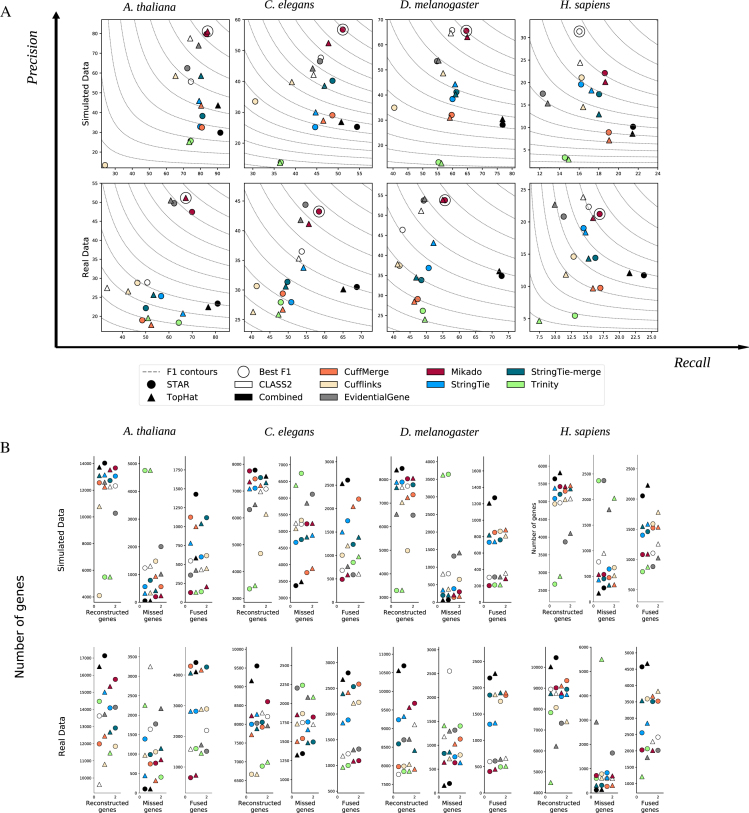
Performance of Mikado on simulated and real data. **(A)** We evaluated the performance of Mikado using both simulated data and the original real data. The method with the best transcript-level F1 is marked by a circle. **(B)** Number of reconstructed, missed, and chimeric genes in each assembly. Notice the lower level of chimeric events in simulated data.

Across the four species and on both simulated and real data, Mikado was able to successfully combine the different assemblies, obtaining a higher accuracy than most individual tools in isolation. Compared with the best overall combination, CLASS2 on STAR alignments, Mikado improved the accuracy by, on average, 6.58% and 9.23% on simulated and real data at the transcript level, respectively (Fig. 3 and Additional File 2). Most of this improvement accrues due to an improved recall rate without significant losses in precision. We register a single exception, on *H. sapiens* simulated data, due to an excess of intronic gene models that pervade the assemblies of all other tools. On simulated data, CLASS2 is able to detect these models and exclude them, most likely using its refined filter on low-coverage regions [12]. However, this increase in precision is absent when TopHat2 is used as an aligner and on real data. While Mikado does not calculate or utilize coverage to score and select transcripts, we do make provision for externally generated metrics that could be used in conjunction with Mikado’s fragment filtering to screen out lowly expressed intronic models. Aside from the accuracy in correctly reconstructing transcript structures, in our experiments, merging and filtering the assemblies proved an effective strategy for producing a comprehensive transcript catalogue. Mikado consistently retrieved more loci than the most accurate tools while avoiding the sharp drop in precision of more sensitive methods such as, e.g., Trinity (Fig. 3B). Finally, Mikado was capable of accurately identifying and solving cases of artifactual gene fusions, which mar the performance of many assemblers. As this kind of error is more prevalent in our real data, the increase in precision obtained by using Mikado was greater using real rather than simulated data.

We further assessed the performance of Mikado in comparison with three other methods that are capable of integrating transcripts from multiple sources: CuffMerge [32], StringTie-merge [15], and EvidentialGene [24, 33]. CuffMerge and StringTie-merge perform a meta-assembly of transcript structures without considering ORFs or homology. In contrast, EvidentialGene is similar to Mikado in that it classifies and selects transcripts, calculating ORFs and associated quality metrics from each transcript to inform its choice. In our tests, Mikado consistently performed better than alternative combiners, in particular, when compared to the two meta-assemblers. The performance of StringTie-merge and CuffMerge on simulated data underscored the advantage of integrating assemblies from multiple sources as both methods generally improved recall over input methods. However, this was accompanied by a drop in precision, most noticeably for CuffMerge, as assembly artifacts present in the input assemblies accumulated in the merged dataset. In contrast, the classification and filtering-based approach of EvidentialGene led to a more precise dataset, but at the cost of a decrease in recall. Mikado managed to balance both aspects, thus showing a better accuracy than any of the alternative approaches (*A. thaliana* +6.24%, *C. elegans* +7.66%, *D. melanogaster* +9.48%, and *H. sapiens* +4.92% F1 improvement over the best alternative method). On real and simulated data, Mikado and EvidentialGene generally performed better than the two meta-assemblers, with an accuracy differential that ranged from moderate in *H. sapiens* (1.67% to 4.32%) to very marked in *A. thaliana* (14.87% to 29.58%). An important factor affecting the accuracy of the meta-assemblers with real data is the prevalence of erroneous transcript fusions that can result from incorrect read alignment, genomic DNA contamination, or bona fide overlap between transcriptional units. Both StringTie-merge and CuffMerge were extremely prone to this type of error, as across the four species they generated, on average, 2.39 times the number of fusion genes compared to alternative methods (Fig. 3B). Between the two selection-based methods, EvidentialGene performed similarly to Mikado on real data but much worse on simulated data. Its accuracy was, on average, 2 points lower than that of Mikado on real data and 8.13 points lower in the simulations. This is primarily due to a much higher precision differential between the two methods in simulated data, with Mikado performing much better than EvidentialGene on this front (+8.95% precision on simulated data).

### Filtering lenient assemblies

**Figure 4:**
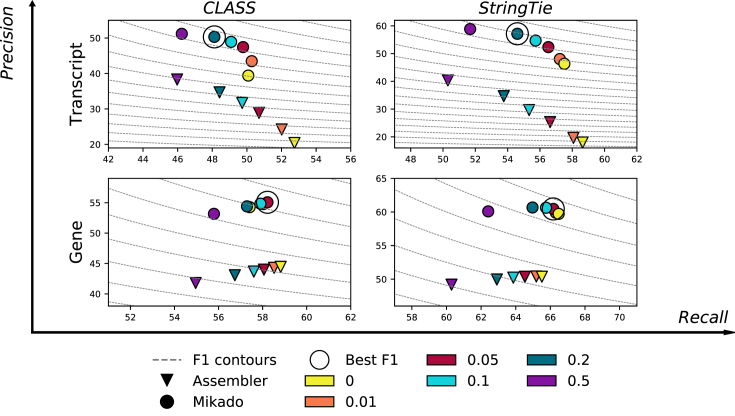
Performance of Mikado while varying the MIF parameter. Precision/recall plot at the gene and transcript levels for CLASS and StringTie at varying minimum isoform fraction thresholds in *A. thaliana*, with and without applying Mikado. Dashed lines mark the F1 levels at different precision and recall values. CLASS sets MIF to 5% by default (red), while StringTie uses a slightly more stringent default value of 10% (cyan).

Although our tests have been conducted using default parameters for the various assemblers, these parameters can be adjusted to alter the balance between precision and sensitivity according to the goal of the experiment. In particular, three of the assemblers we tested provide a parameter to filter out alternative isoforms with a low abundance. This parameter is commonly referred to as “minimum isoform fraction,” or MIF, and sets for each gene a minimum isoform expression threshold relative to the most expressed isoform. Only transcripts whose abundance ratio is greater than the MIF threshold are reported. Therefore, lowering this parameter will yield a higher number of isoforms per locus, retaining transcripts that are expressed at low levels and potentially increasing the number of correctly reconstructed transcripts. This improved recall is obtained at the cost of a drop in precision, as more and more incorrect splicing events are reported (Fig.4). Mikado can be applied on top of these very permissive assemblies to filter out spurious splicing events. In general, filtering with Mikado yielded transcript datasets that are more precise than those produced by the assemblers at any level of chosen MIF or even when comparing the most relaxed MIF in Mikado with the most conservative in the raw assembler output (Fig.4).

### Multisample transcript reconstruction

Unraveling the complexity of the transcriptome requires assessing transcriptional dynamics across many samples. Projects aimed at transcript discovery and genome annotation typically utilize datasets generated across multiple tissues and experimental conditions in order to provide a more complete representation of the transcriptional landscape. Even if a single assembly method is chosen, there is often a need to integrate transcript assemblies constructed from multiple samples. StringTie-merge, CuffMerge, and the recently published TACO [34] have been developed with this specific purpose in mind. The meta-assembly approach of these tools can reconstruct full-length transcripts when they are fragmented in individual assemblies but, as observed earlier, it is prone to creating fusion transcripts. TACO directly addresses this issue with a dedicated algorithmic improvement, i.e., change-point detection. This solution is based on fusion transcripts showing a dip in read coverage in regions of incorrect assembly; this change in coverage can then be used to identify the correct breakpoint. A limitation of the implementation in TACO is that it requires expression estimates to be encoded in the input GTFs, and some tools do not provide this information.

**Figure 5:**
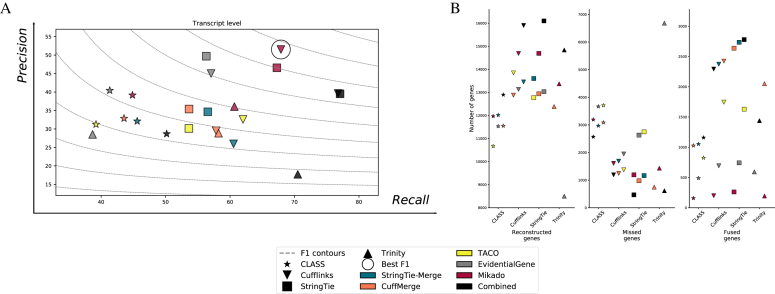
Integrating assemblies coming from multiple samples. **(A)** Mikado performs consistently better than other merging tools. StringTie-merge and TACO are not compatible with Trinity results and as such have not been included in the comparison. **(B)** Rate of recovered, missed, and fused genes for all the assembler and combiner combinations.

To assess the performance of Mikado for multisample reconstruction, we individually aligned and assembled the 12 *A. thaliana* seed development samples from PRJEB7093, using the four single-sample assemblers described previously. The collection of 12 assemblies per tool was then integrated into a single set of assemblies using different combiners. StringTie-merge and TACO could not be applied to the Trinity dataset, as they both require embedded expression data in the GTF files, which is not provided in the Trinity output. In line with the results published in the TACO article [34], we observed a high rate of fusion events in both StringTie-merge and CuffMerge results (Fig. 5B), which TACO reduced. However, none of these tools performed as well as EvidentialGene or Mikado, either in terms of accuracy or in avoiding gene fusions (Fig. 5). Mikado achieved the highest accuracy irrespective of the single sample assembler used, with an improvement in F1 over the best alternative method of +8.25% for Cufflinks assemblies, +2.23% for StringTie, +0.95% for CLASS2, and +6.65% for Trinity.

Transcript assemblies are commonly incorporated into evidence-based gene finding pipelines, often in conjunction with other external evidence such as cross species protein sequences, proteomics data, or synteny. The quality of transcript assembly can therefore potentially have an impact on downstream gene prediction. To test the magnitude of this effect, we used the data from these experiments on *A. thaliana* to perform gene prediction with the popular MAKER annotation pipeline, using Augustus with default parameters for the species as a gene predictor. Our results (Supplementary Fig. S6) show that, as expected, an increased accuracy in the transcriptomic dataset leads to an increased accuracy in the final annotation. Importantly, MAKER was not capable of reducing the prevalence of gene fusion events present in the transcript assemblies. This suggests that *ab initio* Augustus predictions utilized by MAKER do not compensate for incorrect fusion transcripts that are provided as evidence and stress the importance of pruning these mistakes from transcript assemblies before performing an evidence-guided gene prediction.

### Expansion to long-read technologies

Short-read technologies, due to their low per-base cost and extensive breadth and depth of coverage, are commonly utilized in genome annotation pipelines. However, like the previous generation of Sanger ESTs, their short size requires the use of sophisticated methods to reconstruct the structure of the original RNA molecules. Third-generation sequencing technologies promise to remove this limitation by generating full-length cDNA sequences. These new technologies currently offer lower throughput and are less cost effective but have, in recent studies, been employed alongside short-read technologies to define the transcriptome of species with large gene content [35, 36].

We tested the complementarity of the two technologies by sequencing two samples of a standard human reference RNA library with the leading technologies for both approaches, Illumina HiSeq for short reads (250 bp, paired-end reads) and the PacBio IsoSeq protocol for long reads. Given the currently higher per-base costs of long-read sequencing technologies, read coverage is usually much lower than for short-read sequencing. We found many genes to be reconstructed by both platforms. However, as expected given the lower sequencing depth, there was a clear advantage for the Illumina dataset on genes with expression lower than 10 Transcripts Per Million (Supplementary Fig. S7). We verified the feasibility of integrating the results given by the different sequencing technologies by combining the long reads with the short-read assemblies, either simply concatenating them or by filtering them with EvidentialGene and Mikado (Supplementary Fig. S8). An advantage of Mikado over the two alternative approaches is that it enables it to prioritize PacBio reads over Illumina assemblies by giving them a slightly higher base score. In this analysis, we saw that even PacBio data on its own might require some filtering, as the original sample contains a mixture of whole and fragmented molecules, together with immature transcripts. Both Mikado and EvidentialGene are capable of identifying mature coding transcripts in the data, but Mikado shows a better recall and general accuracy rate, albeit at the cost of some precision. However, Mikado performed much better than EvidentialGene in filtering either the Illumina data on its own or the combination of the two technologies. Although the filtering inevitably loses some of the real transcripts, the loss is compensated by an increased overall accuracy. Mikado performed better than EvidentialGene in this respect, as the latter did not noticeably improve in accuracy when given a combination of PacBio and Illumina data, rather than the Illumina data alone.

## Conclusions

Transcriptome assembly is a crucial component of genome annotation workflows; however, correctly reconstructing transcripts from short RNA-seq reads remains a challenging task. In recent years, methods for both *de novo* and reference-guided transcript reconstruction have accumulated rapidly. When combined with the large number of RNA-seq mapping tools, deciding on the optimal transcriptome assembly strategy for a given organism and RNA-seq dataset (stranded/unstranded, polyA/ribodepleted) can be bewildering. Here, we showed that different assembly tools are complementary to each other, fully reconstructing genes only partially reconstructed or missing entirely from alternative approaches. Similarly, when analyzing multiple RNA-seq samples, the complete transcript catalogue is often only obtained by collating together different assemblies. For a gene annotation project, it is therefore typical to have multiple sets of transcripts, be they derived from alternative assemblers, different assembly parameters, or arising from multiple samples. Our tool, Mikado, provides a framework for integrating transcript assemblies, exploiting the inherent complementarity of the data to produce a high-quality transcript catalogue. As Mikado is capable of accepting data from multiple standard file formats (GFF3, BED12, GTF), its applications are wider than those presented in this article. Although it is not discussed fully here, the Daijin pipeline already supports additional aligners and assemblers, such as Scallop [37] or HISAT2 [8]. Similar to what we have shown in this manuscript, Mikado can be fruitfully applied to assembly workflows based on these tools (Supplementary Fig. S9); as such, it provides a mechanism to integrate transcript assemblies from both new and established methods.

Rather than attempting to capture all transcripts, our approach aims to mimic the selective process of manual curation by evaluating and identifying a subset of transcripts from each locus. The criteria for selection can be configured by the user, enabling them to, e.g., penalize gene models with truncated ORFs, those with noncanonical splicing, targets for nonsense mediated decay, or chimeric transcripts spanning multiple genes. Such gene models may represent bona fide transcripts (with potentially functional roles) but can also arise from aberrant splicing or, as seen from our simulated data, from incorrect read alignment and assembly. Mikado acts as a filter principally to identify coding transcripts with complete ORFs and is therefore in line with most reference annotation projects that similarly do not attempt to represent all transcribed sequences. Our approach is made possible by integrating the data on transcript structures with additional information generally not utilized by transcript assemblers such as similarity to known proteins, the location of ORFs, and information on the reliability of splicing junctions. This information aids Mikado in performing operations such as discarding spurious alternative splicing events or detecting chimeric transcripts. This allows Mikado to greatly improve precision over the original assemblies, with, in general, minimal drops in recall. Moreover, similar to TACO, Mikado is capable of identifying and resolving chimeric assemblies, which negatively affect the precision of many of the most sensitive tools, such as StringTie or the two meta-assemblers Cuffmerge and StringTie-merge.

Genome annotation involves making choices about what genes and transcripts to include in the gene set, and different annotators will make different choices dependent on their own motivations and available data. The manually annotated genomes of human and *A. thaliana* exhibit clear differences. The annotation of the human genome is very comprehensive, with an average of five transcripts per gene. In contrast, the TAIR10 annotation of *A. thaliana* captures fewer splice variants, with most genes being annotated with a single, coding isoform. This reflects not only potentially real differences in the extent of alternative splicing between the species but also differences in the annotation approach, with the human gene set capturing, in addition to coding splice variants, transcripts lacking annotated ORFs and those with retained introns or otherwise flagged as targets for nonsense mediated decay. Neither the more comprehensive nor the more conservative approach is necessarily the most correct. The purpose of the annotation, i.e., how it will be used by the research community, and the types of supporting data will guide the selection process. Mikado provides a framework to apply different selection criteria, therefore similar to *ab initio* programs where the results are heavily dependent on the initial training set. Also for Mikado, the results will depend on the experimenter’s choices. In the online documentation, we provide a discussion on how to customize scoring files according to the needs of the experimenter and a tutorial to guide through its creation [31].

Our experiments show that Mikado can aid genome annotation by generating a set of high-quality transcript assemblies across a range of different scenarios. Rather than having to identify the best aligner/assembly combination for every project, Mikado can be used to integrate assemblies from multiple methods, with our approach reliably identifying the most accurate transcript reconstructions and allowing the user to tailor the gene set to their own requirements. It is also simple to incorporate assemblies from new tools, even if the new method is not individually the most accurate approach. Given the challenges associated with short-read assembly, it is desirable (when available) to integrate these with full-length cDNA sequences. Mikado is capable of correctly integrating analyses coming from different assemblers and technologies, including mixtures of Illumina and PacBio data. Our tool has already been employed for such a task on the large, repetitive genome of *Triticum aestivum* [36], where it was instrumental in selecting a set of gene models from more than 10 million transcript assemblies and PacBio IsoSeq reads. The consolidated dataset returned by Mikado was almost 30 times smaller than the original input dataset, and this polishing was essential to both ensure a high-quality annotation and to reduce the running times of downstream processes.

In conclusion, Mikado is a flexible tool that is capable of handling a plethora of data types and formats. Its novel selection algorithm was shown to perform well in model organisms and was central in the genome annotation pipeline of various species [36, 38, 39]. Its deployment should provide genome annotators with another powerful tool to improve the accuracy of data for subsequent *ab initio* training and evidence-guided gene prediction.

## Methods

### Input datasets

For *C. elegans*, *D. melanogaster*, and *H. sapiens*, we retrieved from the European Nucleotide Archive (ENA) the raw reads used for the evaluation in [19], under Bioproject PRJEB4208. We further selected and downloaded a publicly available strand-specific RNA-seq dataset for *A. thaliana*, PRJEB7093. Congruent with the assessment in [19], we used genome assemblies and annotations from EnsEMBL v. 70 for all metazoan species, while for *A. thaliana* we used the TAIR10 version. For all species, we simulated reads using the input datasets as templates. Reads were trimmed with TrimGalore v0.4.0 [40] and aligned onto the genome with Bowtie v1.1.2 [41] and HISAT v2.0.4 [8]. The HISAT alignments were used to calculate the expression levels for each transcript using Cufflinks v2.2.1, while the Bowtie mappings were used to generate an error model for the SPANKI Simulator v.0.5.0 [42]. The transcript coverages and the error model were then used to generate simulated reads, at a depth of 10X for *C. elegans* and *D. melanogaster* and 3X for *A. thaliana* and *H. sapiens*. A lower coverage multiplier was applied to the latter species to have a similar number of reads for all four datasets, given the higher sequencing depth in the *A. thaliana* dataset and the higher number of reference transcripts in H. sapiens. cDNA sequences for *A. thaliana* were retrieved from the National Center for Biotechnology Information (NCBI) nucleotide database on 21 April 2017, using the query:

“Arabidopsis” [Organism] OR arabidopsis[All Fields]) AND “Arabidopsis thaliana” [porgn] AND biomol\_mrna [PROP]

For the second experiment on *H. sapiens*, we sequenced two samples of the Stratagene Universal Human Reference RNA (catalogue ID 740000), which consists of a mixture of RNA derived from 10 cell lines. One sample was sequenced on an Illumina HiSeq2000 and the second on a PacBio RSII machine. Sequencing runs were deposited in ENA, under the project accession code PRJEB22606.

#### Preparation and sequencing of Illumina libraries

The libraries for this project were constructed using the NEXTflex Rapid Directional RNA-seq Kit (PN: 5138-08) with the NEXTflex DNA Barcodes - 48 (PN: 514104) diluted to 6  μm. The library preparation involved an initial quality check of the RNA using Qubit DNA (Life Technologies Q32854) and RNA (Life Technologies Q32852) assays as well as a quality check using the PerkinElmer GX with the RNA assay (PN:CLS960010)

Then, 1  μg of RNA was purified to extract mRNA with a poly-A pull-down using biotin beads; fragmented and first-strand cDNA was synthesized. This process reverse transcribes the cleaved RNA fragments primed with random hexamers into first-strand cDNA using reverse transcriptase and random primers. The second-strand synthesis process removes the RNA template and synthesizes a replacement strand to generate dscDNA. The ends of the samples were repaired using the 3′ to 5′ exonuclease activity to remove the 3′ overhangs and the polymerase activity to fill in the 5′ overhangs, creating blunt ends. A single ‘A’ nucleotide was added to the 3′ ends of the blunt fragments to prevent them from ligating to one another during the adapter ligation reaction. A corresponding single ‘T’ nucleotide on the 3′ end of the adapter provided a complementary overhang for ligating the adapter to the fragment. This strategy ensured a low rate of chimera formation. The ligation of a number indexing adapters to the ends of the DNA fragments prepared them for hybridization onto a flow cell. The ligated products were subjected to a bead-based size selection using Beckman Coulter XP beads (PN: A63880). In addition to performing a size selection, this process removed the majority of unligated adapters. Prior to hybridization to the flow cell, the samples were subjected to polymerase chain reaction (PCR) to enrich for DNA fragments with adapter molecules on both ends and to amplify the amount of DNA in the library. Directionality is retained by adding deoxyuridine triphosphate during the second strand synthesis step and subsequent cleavage of the uridine-containing strand using uracil DNA glycosylase. The strand that was sequenced is the cDNA strand. The insert size of the libraries was verified by running an aliquot of the DNA library on a PerkinElmer GX using the high-sensitivity DNA chip (PerkinElmer CLS760672); the concentration was determined using a high-sensitivity Qubit assay and q-PCR.

The constructed stranded RNA libraries were normalized and equimolar pooled into two pools. The pools were quantified using a KAPA Library Quant Kit Illumina/ABI (KAPA KK4835) and found to be 6.71  nm and 6.47  nm, respectively. A 2- nm dilution of each pool was prepared with NaOH at a final concentration of 0.1 N and incubated for 5 minutes at room temperature to denature the libraries. Then, 5  μL of each 2- nm dilution was combined with 995  μL HT1 (Illumina) to give a final concentration of 10  pm; 135  μL of the diluted and denatured library pool was then transferred into a 200- μL strip tube, spiked with 1% PhiX Control v3 (Illumina FC-110-3001), and placed on ice before loading onto the Illumina cBot with a Rapid v2 Paired-end flow cell and HiSeq Rapid Duo cBot Sample Loading Kit (Illumina CT-403-2001). The flow cell was loaded on a HiSeq 2500 (rapid mode) following the manufacturer’s instructions with a HiSeq Rapid SBS Kit v2 (500 cycles) (Illumina FC-402-4023) and HiSeq PE Rapid Cluster Kit v2 (Illumina PE-402-4002). The run setup was as follows: 251 cycles/7 cycles(index)/251 cycles utilizing HiSeq Control Software 2.2.58 and RTA 1.18.64. Reads in .bcl format were demultiplexed based on the 6-bp Illumina index by CASAVA 1.8 (Illumina), allowing for a one base-pair mismatch per library, and converted to FASTQ format by bcl2fastq (Illumina).

#### Preparation and sequencing of PacBio libraries

The Iso-Seq libraries were created starting from 1  μg of human total RNA; full-length cDNA was then generated using the SMARTer PCR cDNA Synthesis Kit (Clontech, Takara Bio Inc., Shiga, Japan) following PacBio recommendations set out in the Iso-Seq method [43]. PCR optimization was carried out on the full-length cDNA using the KAPA HiFi PCR Kit (Kapa Biosystems, Boston, Massachusetts, USA); 12 cycles were sufficient to generate the material required for ELF size selection. A timed setting was used to fractionate the cDNA into 12 individual-sized fractions using the SageELF (Sage Science Inc., Beverly, Massachusetts, USA) on a 0.75% ELF Cassette. Prior to further PCR, the ELF fractions were equimolar pooled into the following sized bins: 0.7–2kb, 2–3kb, 3–5kb, and > 5kb. PCR was repeated on each bin to generate enough material for SMRTbell library preparation; this was completed following PacBio recommendations in the Iso-Seq method. The four libraries generated were quality checked using Qubit Florometer 2.0 and sized using the Bioanalyzer HS DNA chip. The loading calculations for sequencing were completed using the PacBio Binding Calculator v2.3.1.1 [44]. The sequencing primer was used from the SMRTbell Template Prep Kit 1.0 and was annealed to the adapter sequence of the libraries. Each library was bound to the sequencing polymerase with the DNA/Polymerase Binding Kit v2. The complex formed was then bound to Magbeads in preparation for sequencing using the MagBead Kit v1. Calculations for primer and polymerase binding ratios were kept at default values. The libraries were prepared for sequencing using the PacBio-recommended instructions laid out in the Binding Calculator. The sequencing chemistry used to sequence all libraries was DNA Sequencing Reagent Kit 4.0; the Instrument Control Software version was v2.3.0.0.140640. The libraries were loaded onto PacBio RS II SMRT Cells 8Pac v3; each library was sequenced on three SMRT Cells. All libraries were run without stage start and 240 minute movies per cell. Reads for the four libraries were extracted using SMRT Pipe v2.3.3, following the manufacturer's instructions [45].

### Alignments and assemblies

Reads from the experiments were aligned using STAR v2.4.1c and TopHat v2.0.14. For STAR, read alignment parameters for all species were as follows:

--outFilterMismatchNmax 4 --alignSJoverhangMin 12 --alignSJDBoverhangMin 12 --outFilterIntronMotifs RemoveNoncanonical --alignEndsType EndToEnd --alignTranscriptsPerReadNmax 100000 --alignIntronMin MININTRON --alignIntronMax MAXINTRON --alignMatesGapMax MAXINTRON

For TopHat2, we used the following parameters:

-r 50 -p 4 --min-anchor-length 12 --max-multihits 20 --library-type fr-unstranded -i MININTRON -I MAXINTRON

The parameters “MINTRON” and “MAXINTRON” were varied for each species, as follows:

- *A. thaliana*: minimum 20, maximum 10,000
- *C. elegans*: minimum 30, maximum 15,000
- *D. melanogaster*: minimum 20, maximum 10,000
- *H. sapiens*: minimum 20, maximum 10,000

Each dataset was assembled using the following four tools: CLASS v 2.12, Cufflinks 2.1.1, StringTie v. 1.03, and Trinity r20140717. Command lines for the tools were as follows:

- CLASS: we executed this tools through a wrapper included in Mikado, class_run.py, with command line parameters -F 0.05
- Cufflinks: -u -F 0.05; for the *A. thaliana* dataset, we further specified –library-type fr-firststrand.
- StringTie: -m 200 -f 0.05
- Trinity: –genome_guided_max_intron MAXINTRON (see above)

Trinity assemblies were mapped against the genome using GMAP v20141229 [46], with parameters -n 0 –min-trimmed-coverage=0.70 –min-identity=0.95. For simulated data, we elected to use a more modern version of Trinity (v.2.3.2), as the older version was unable to assemble transcripts correctly for some of the datasets. For assembling separately the samples in PRJBE7093, we used Cufflinks (v.2.2.1) and StringTie v1.2.3 with default parameters.

### Mikado analyses

All analyses were run with Mikado 1.0.1, using Daijin to drive the pipeline. For each species, we built a separate reference protein dataset to be used for the BLAST comparison (see Supplementary Table S4). We used NCBI BLASTX v2.3.0 [47], with a maximum e-value of 10e-7 and a maximum number of targets of 10. ORFs were predicted using TransDecoder 3.0.0 [10]. Scoring parameters for each species can be found in Mikado v1.0.1 [48], with a name scheme of *species_name*_scoring.yaml (e.g., “athaliana_scoring.yaml” for *A. thaliana*). The same scoring files were used for all runs, both with simulated and real data. Filtered junctions were calculated using Portcullis v1.0 beta5, using default parameters.

Mikado was instructed to look for models with, among other features, a good UTR/CDS proportion (adjusted per species), homology to known proteins, and a high proportion of validated splicing junctions. We further instructed Mikado to remove transcripts that do not meet minimum criteria, such as having at least a validated splicing junction if any is present in the locus and a minimum transcript length or CDS length. The configuration files are bundled with the Mikado software as part of the distribution.

### Details on the algorithms of Mikado

The Mikado pipeline is divided into three distinct phases, described below.

#### Mikado prepare

*Mikado prepare* is responsible for bringing together multiple annotations into a single GTF file. This step of the pipeline is capable of handling both GTF and GFF3 files, making it adaptable to use data from most assemblers and cDNA aligners currently available. *Mikado prepare* will not just make the data format uniform but will also perform the following operations:

- It will optionally discard any model below a user-specified size (default 200 base pairs).
- It will analyze the introns present in each model and verify their canonicity. If a model is found to contain introns from both strands, it will be discarded by default, although the user can decide to override this behavior and keep such models in. Each multiexonic transcript will be tagged with this information, making it possible for Mikado to understand the number of canonical splicing events present in a transcript later on.
- Mikado will also switch the strand of multiexonic transcripts if it finds that their introns are allocated to the wrong strand, and it will strip the strand information from any monoexonic transcript coming from nonstrand-specific assemblies
- Finally, Mikado will sort the models, providing a coordinate-ordered GTF file as output, together with a FASTA file of all the cDNAs that have been retained.

*Mikado prepare* uses temporary SQLite databases to perform the sorting operation with a limited amount of memory. As such, it is capable of handling millions of transcripts from multiple assemblies with the memory found on a regular modern desktop PC (less than 8 GB of random access memory).

#### Mikado serialize

*Mikado serialize* is the part of the pipeline whose role is to collect all additional data on the models and store it into a standard database. Currently, Mikado is capable of handling the following types of data:

- FASTAs, i.e., the cDNA sequences produced by *Mikado prepare*, and the genome sequence.
- Genomic BED files containing the location of trusted introns. Usually these are either output directly from the aligners themselves (e.g., the “junctions.bed” file produced by TopHat) or derived from the alignment using a specialized program such as Portcullis.
- Transcriptomic BED or GFF3 files containing the location of the ORFs on the transcripts. These can be calculated with any program chosen by the user. We highly recommend using a program capable of indicating more than one ORF per transcript if more than one is present, as Mikado relies on this information to detect and solve chimeric transcripts. Both TransDecoder and Prodigal have such capability.
- Homology match files in XML format. These can be produced either by BLAST+ or by DIAMOND (v 0.8.7 and later) with the option “-outfmt 5.”

*Mikado serialize* will try to keep the memory consumption at a minimum by limiting the amount of maximum objects present in memory (the threshold can be specified by the user, with the default being at 20,000). XML files can be analyzed in parallel, so *Mikado serialize* can operate more efficiently if BLAST or DIAMOND runs are performed by prechunking the cDNA FASTA file and producing corresponding multiple output files.

*Mikado serialize* will output a database with the structure shown in Supplementary Fig. S10.

#### Mikado pick

*Mikado pick* selects the final transcript models and outputs them in GFF3 format. In contrast with many *ab initio* predictors, currently Mikado does not provide an automated system to learn the best parameters for a species. Rather, the choice of what types of models should be prioritized for inclusion in the final annotation is left to the experimenter, depending on his or her needs and goals. For the experiments detailed in this article, we configured Mikado to prioritize complete protein-coding models and to apply only a limited upfront filtering to transcripts. A stricter upfront hard-filtering of transcripts, e.g., involving discarding any monoexonic transcript without sufficient homology support, might have yielded a more precise collated annotation at the price of discarding any potentially novel monoexonic genes. Although we provide the scoring files used for this article in the software distribution, we encourage users to inspect them and adjust them to their specific needs. As part of the workflow, Mikado also produces tabular files, with all the metrics calculated for each transcript, and the relative scores. It is therefore possible for the user to use this information to adjust the scoring model. The GFF3 files produced by Mikado comply with the formal specification of GFF3, as defined by the sequence ontology and verified using GenomeTools v.1.5.9 or later. Earlier versions of GenomeTools would not validate Mikado files completely due to a bug in their calculation of CDS phases for truncated models, see issue 793 on GenomeTools GitHub [49].

### Integration of multiple transcript assemblies

Evidential Gene v20160320 [24] was run with default parameters in conjunction with CDHIT v4.6.4 [50]. Models selected by the tools were extracted from the combined GTFs using a mikado utility, mikado grep, and further clustered into gene loci using gffread from Cufflinks v2.2.1. StringTie-merge and Cuffmerge were run with default parameters. Limited to the experiment regarding the integration of assemblies from multiple samples, we used TACO v0.7. For all three tools, we used their default isoform fraction parameter. The GTFs produced by the TACO meta-assemblies were reordered using a custom script (“sort_taco_assemblies.py”) present in the script repository.

### MAKER runs

We used MAKER v2.31.8 [51] in combination with Augustus 3.2.2 [52] for all our runs. GFFs and GTFs were converted to a match/match_part format for MAKER using the internal script of the tool “cufflinks2gff3.pl.” MAKER was run using Message Passing Interface (MPI) and default parameters; the only input files were the different assemblies produced by the tested tools.

### Comparison with reference annotations

All comparisons have been made using Mikado compare v1.0.1. Briefly, Mikado compare creates an interval tree structure of the reference annotation, which is used to find matches in the vicinity of any given prediction annotation. All possible matches are then evaluated in terms of nucleotide, junction and exonic recall, and precision; the best one is reported as the best match for each prediction in a transcript map (TMAP) file. After exhausting all possible predictions, Mikado reports the best match for each reference transcript in the “reference map” (REFMAP) file and general statistics about the run in a statistics file. Mikado compare is capable of detecting fusion genes in the prediction, defined as events where a prediction transcript intersects at least one transcript per gene from at least two different genes, with either a junction in common with the transcript or an overlap over 10% of the length of the shorter between the prediction or the reference transcripts. Fusion events are reported using a modified class code, with a “f,” prepending it. For a full introduction to the program, we direct the reader to the online documentation [53].

#### Creation of reference and filtered datasets for the comparisons

For *A. thaliana*, we filtered the TAIR10 GFF3 to retain only protein coding genes. For the other three species, reference GTF files obtained through EnsEMBL were filtered using the “clean_reference.py” python script present in the “Assemblies” folder of the script repository (see the Script availability section). The YAML configuration files used for each species can be found in the Biotypes folder. The retained models constitute our reference transcriptome for comparisons.

For all analyses, we deemed a transcript reconstructable if all of its splicing junctions (if any) and all of its internal bases could be covered by at least one read. As read coverage typically decreases or disappears at the end of transcripts, we used the mikado utility “trim” to truncate the terminal UTR exons until their lengths reached the maximum allowed value (50 bps for our analysis) or the beginning of the CDS section is found. BEDTools v. 2.27 beta (commit 6114307 [54]) was then used to calculate the coverage of each region. Detected junctions were calculated using Portcullis, specifically using the BED file provided at the end of the Portcullis junction analysis step. The “get_filtered_reference.py” was then used to identify reconstructable transcripts.

For simulated datasets, we used the BAM file provided by SPANKI to derive the list of reconstructable transcripts. For the nonsimulated datasets, we used the union of transcripts found to be reconstructable from each of the alignment methods. The utility “mikado util grep” was used to extract the relevant transcripts from the reference files. Details of the process can be found in the two snakemakes “compare.snakefile” and “compare_simulations.snakefile” present in the “Snakemake” directory of the script repository.

#### Calculation of comparison statistics

“Mikado compare” was used to assess the similarity of each transcript set against both the complete reference and the reference filtered for reconstructable transcripts. Precision statistics were calculated from the former, while recall statistics were calculated from the latter.

### Customization and further development

Mikado make it possible to customize its run mode through the use of detailed configuration files. There are two basic configuration files: one is dedicated to the scoring system and the other contains run-specific details. The scoring file is divided in four sections and allows the user to specify which transcripts should be filtered out at any of the stages during picking and how to prioritize transcripts through a scoring system. Details on the metrics and on how to write a valid configuration file can be found in the supplementry infomation and the online documentation [58]. These configuration files are intended to be used across runs, akin to how standard parameter sets are re-used in *ab initio* gene prediction programs, e.g., Augustus. The second configuration file contains parameters pertaining to each run, such as the position of the input files, the type of database to be used, or the desired location for output files. As such, they are meant to be customized by the user for each experiment. Mikado provides a command, “mikado configure,” that will generate this configuration file automatically when given basic instructions.

## Availability of source code and requirements

- Project name: Mikado
- Project home page: [1]
- Operating system(s): Linux
- Programming language: Python3
- Other requirements: SnakeMake, BioPython, NumPY, SciPY, Scikit-learn, BLAST+ or DIAMOND, Prodigal or TransDecoder, Portcullis
- Available through: PyPI, bioconda, SciCruch (RRID: SCR_016159)
- License: GNU LGPL3

## Availability of supporting data

The datasets supporting the conclusions presented in this article are included within the article (and its additional files). Transcript assemblies and gene annotation produced during the current study are available in the *GigaScience* Database [57] and in FigShare [56] together with the source code of the version of our software tool used to perform all experiments in this study. The sequencing runs analyzed for this article can be found on ENA, under the accession codes PRJEB7093 (for *A. thaliana*) and PRJEB4028 (for the other three species). The human sequencing data of our parallel Illumina and PacBio experiment can be found under the accession code PRJEB22606. Mikado is present on GitHub [1]. Many of the scripts used to control the pipeline executions, together with the scripts used to create the charts presented in the article, can be found in the complementary repository [55]. Extensive documentation for the program is available in the “docs” folder in the GitHub repository [1] and is published on the “Read The Docs” website [26]. All sequencing runs and reference sequence datasets used for this study are publicly available. Please see the section “Input datasets” for details. Scripts and configuration files used for the analyses can be found on GitHub [55], in FigShare [56] and in the *GigaScience* Database [57].

## Additional files

Additional file 1 — Supplemental Information

Additional information for the main article, including supplemental figures and tables.

Additional file 2 — Reconstruction statistics for the input methods

This Excel file contains precision, recall and F1 statistics for the various methods tested.

## Abbreviations

BBSRC: Biotechnology and Biological Sciences Research Council; BLAST: Basic Local Alignment Search Tool; CDS: coding sequence; dUTP: deoxyuridine triphosphate; ENA: European Nucleotide Archive; EST: Expressed Sequence Tag; GFF: General Feature Format; GTF: General Transfer Format; MIF: minimum isoform fraction; MPI: Message Passing Interface; NCBI: National Center for Biotechnology Information; ORF: open reading frame; PacBio: Pacific Biosciences; PCR: polymerase chain reaction; RNA-seq: RNA sequencing; UTR: untranslated region

## Competing interests

The authors declare that they have no competing interests.

## Funding

This work was strategically funded by the Biotechnology and Biological Sciences Research Council (BBSRC), Core Strategic Programme (grant BB/CSP1720/1) at the Earlham Institute and by a strategic longer and larger award (BB/J003743/1). Next-generation sequencing and library construction were delivered via the BBSRC National Capability in Genomics (BB/CCG1720/1) at Earlham Institute by members of the Genomics Pipelines Group.

## Author contributions

The lead author of this manuscript is L.V. We describe contributions for all authors to this article using the CRediT taxonomy. The order of authors for each task represents their relative contribution. Conceptualization: D.S. and L.V.; methodology: L.V. and D.S.; software: L.V. and D.M.; validation: L.V., S.C., G.K., and D.S.; writing the original draft: L.V. and D.S.; final writing, reviewing, and editing: L.V., D.S., D.M., S.C., and G.K.; visualization: L.V. and S.C.; and supervision: D.S.

## Supplementary Material

GIGA-D-18-00017_Original_Submission.pdf

GIGA-D-18-00017_Revision_1.pdf

GIGA-D-18-00017_Revision_2.pdf

Response_to_Reviewer_Comments_Original_Submission.pdf

Response_to_Reviewer_Comments_Revision_1.pdf

Reviewer_1_Report_(Original_Submission) -- Simon van Heeringen, Ph.D. 13 Feb 2018 Reviewed

Reviewer_2_Report_(Original_Submission) -- Adam Freedman, Ph.D. 2/28/2018 Reviewed

Supplemental Files
