# Supplementary material for: Leveraging multiple transcriptome assembly methods for improved gene structure annotation
Source: Gigascience. 2018 Jul 24;7(8):giy093. doi: 10.1093/gigascience/giy093 (PMC6105091; doi:10.1093/gigascience/giy093)
Supplement: Supplemental Files [file giy093_supplemental_files.zip › supplemental-diff47ad17.pdf]

Tables

	A. thaliana	*C. elegans*	*D. melanogaster*	*H. sapiens*
Number of genes	27416	43277	14475	37214
Number of coding genes	27397	20517	13923	23249
Number of monoexonic genes	5926	23212	2408	2891
Number of transcripts	35386	53999	27643	184770
Transcripts per gene (average)	1.29	1.25	1.91	4.97
Exons per transcript (average)	5.86	4.26	5.81	6.78
Exon length (average)	261.76	215.58	475.72	233.16
Intron length (average)	164.68	351.13	1570.72	6169.78
Intergenic distances (average)	2159.72	846.18	3268.02	36531.92

Table ST1: Features of the reference annotations

Number of methods	*A. thaliana*		*C. elegans*		*D. melanogaster*		*H. sapiens*		Average
0	869	4.69%	2157	17.45%	474	4.15%	1251	10.16%	9.11%
1	1019	5.49%	789	6.38%	350	3.06%	762	6.19%	5.28%
2	949	5.12%	566	4.58%	605	5.29%	690	5.61%	5.15%
3	1131	6.10%	625	5.06%	483	4.22%	736	5.98%	5.34%
4	1480	7.98%	703	5.69%	996	8.71%	905	7.35%	7.43%
5	2099	11.32%	793	6.42%	843	7.37%	895	7.27%	8.09%
6	2952	15.92%	1361	11.01%	1869	16.34%	1576	12.80%	14.02%
7	3896	21.01%	1685	13.63%	1776	15.53%	3073	24.97%	18.78%
8	4150	22.38%	3682	29.79%	4039	35.32%	2421	19.67%	26.79%

Table ST2: Number of methods capable of reconstructing a given transcript, in all input species.

Category	Description	Example metrics	Number of metrics
**Descriptive**	These metrics merely provide a description of the transcript (eg. its ID) and are not used for scoring.	*tid*, *gid*	3
**cDNA**	These metrics refer to basic features of any transcript such as its number of exons or its cDNA length.	*cdna_length*, *exon_num*	3
**CDS**	These metrics refer to features related to the CDS assigned to the transcript.	*is_complete*, *selected_cds_length*, *number_internal_orfs*	24
**Intron**	These metrics refer to features related to the number of introns and their lengths.	*canonical_intron_proportion*, *suspicious_splicing*	7
**Locus**	These metrics refer to features of the transcript in relationship to all other transcripts in its locus, eg how many of the introns present in the locus are present in the transcript. These metrics are calculated by Mikado during the picking phase, and as such their value can vary during the different stages as the transcripts are shifted to different groups.	*exon_fraction*, *retained_fraction*	8
**UTR**	These metrics refer to features related to the UTR of the transcript. In the case in which a transcript has been assigned multiple ORFs, unless otherwise stated the UTR metrics will be derived only considering the *selected* ORF, not the combination of all of them.	*five_utr_num*, *utr_fraction*	11
**External**	These metrics are derived from accessory data that is recovered for the transcript during the run time. Examples include data regarding the number of introns confirmed by external programs such as PortCullis, or the BLAST score of the best hits.	*blast_score*, *verified_introns_num*	11

Table ST3: Available metrics for scoring transcripts

Target	Species	Version	Source
A. thaliana	*Arabidopsis lyrata*	v1.0	Phytozome v11 [1, 2, 3]
	Brassica rapa	v1.3	Phytozome v11 [1, 2, 4]
	Capsella grandiflora	v1.1	Phytozome v11 [1, 2, 5]
C. elegans	*Caenhorabditis brenneri*	WS251	WormBase [6]
	Caenhorabditis briggsae	WS251	WormBase [6]
D. melanogaster	*Anopheles gambiae*	P4.30	EnsEMBL Metazoa release 30 [7]
	Aedes aegypti	L3.30	EnsEMBL release 30 [7]
	Drosophila ananassae	1.30	EnsEMBL release 30 [7]
H. sapiens	*Canis familiaris*	3.1	EnsEMBL release 83 [8]
	Mus musculus	GRCm38	EnsEMBL release 83 [8]
	Rattus norvegicus	6.0	EnsEMBL release 83 [8]

Table ST4: Protein sequences used as reference for BLAST analyses.

Figures

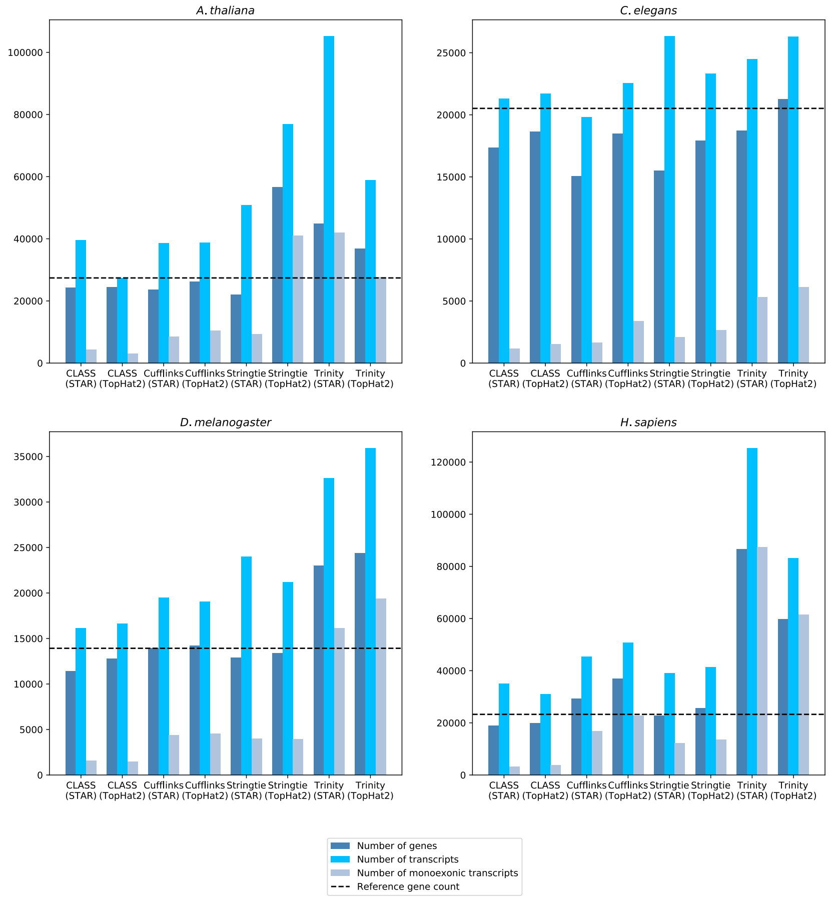

Figure SF1: ~~Assembly statistics~~ Number of genes and transcripts assembled for each method and species. The histogram shows the number of predicted transcripts and genes for each method, while the dashed line reports the true number of gene presents in the reference annotation, for comparison.

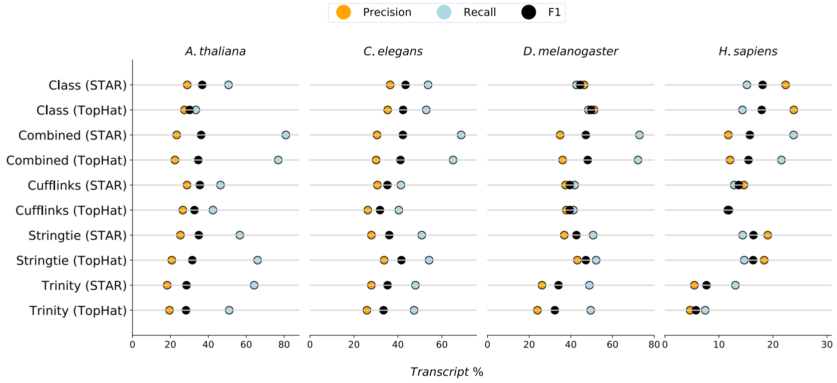

Figure SF2: Accuracy of the input methods when compared to the reference annotations.

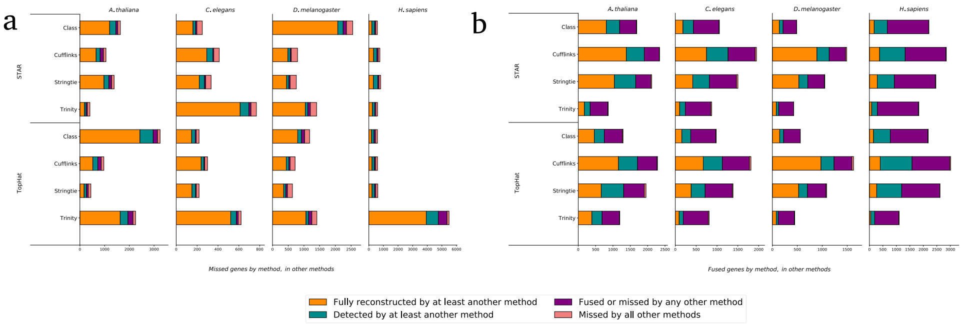

Figure SF3: Genes were divided according to their best class code in the REFMAP file. Fully reconstructed genes fall within the “Match” category, missed genes within the “Fragment”, “Intronic” and “Unknown” category; all other categories are grouped under the “detected” label. a A common error is for an assembler to not reconstruct a gene which is expressed in the sample. Trinity and CLASS were particularly prone to this kind of error. Note that in *C.elegans*, all assemblers neglected to reconstruct large numbers of small ncRNAs, leading to an inflated count of missing genes. For this reason, for this species we only plot genes that have been fully reconstructed by at least one of the possible combinations of aligner and assembler. b Different combinations of aligner and assembler are more or less prone to produce artefactual chimeras. In our experiments, StringTie and Cufflinks produced a large number of such artefacts, even while other methods on the same data were capable of reconstructing genes correctly.

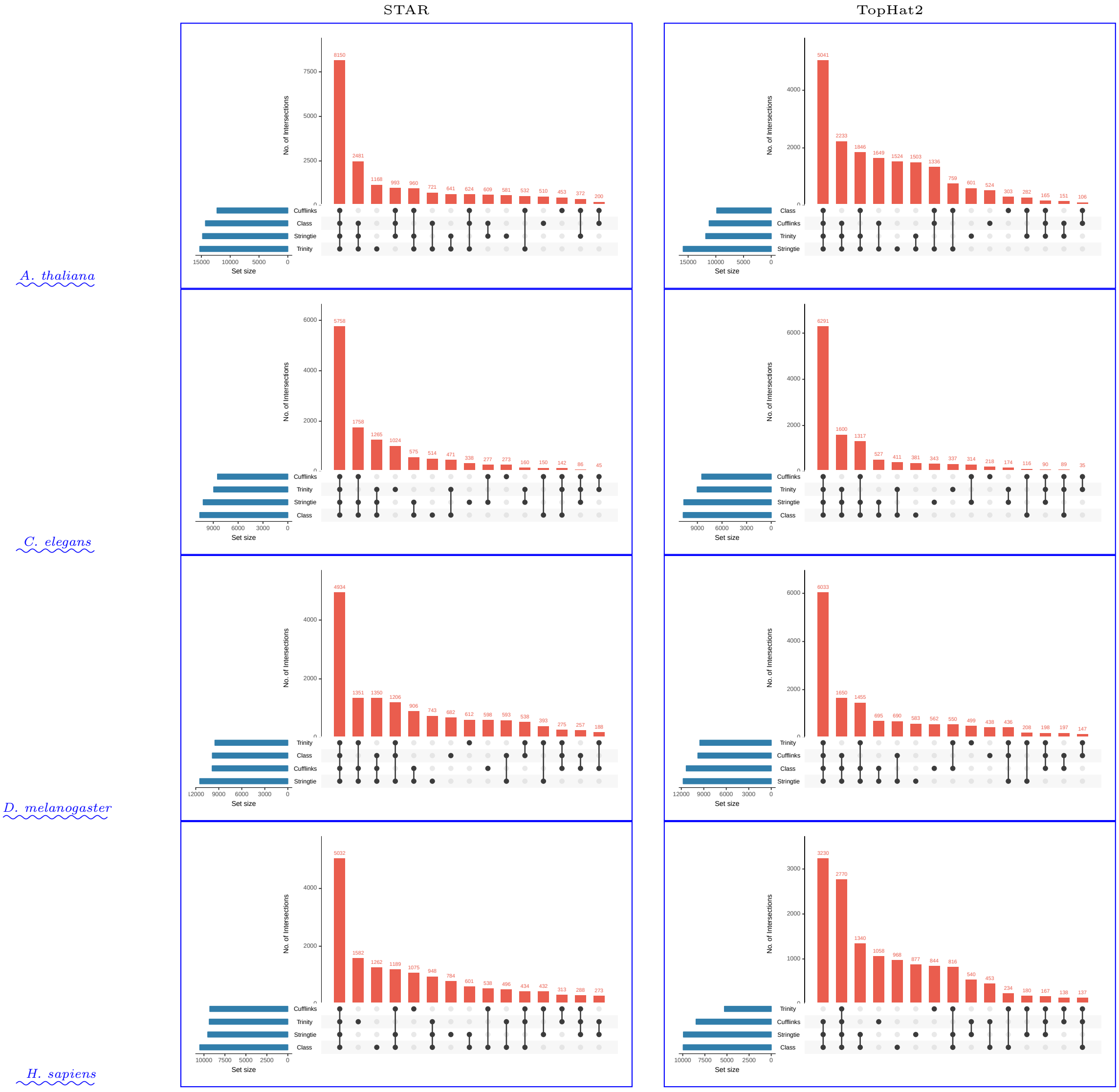

Figure SF4: UpSet plot [9, 10] representing how many transcripts were reconstructed correctly by each assembler. Each tool proved itself to be capable of retrieving transcripts missed by other methods, with varying results in different species.

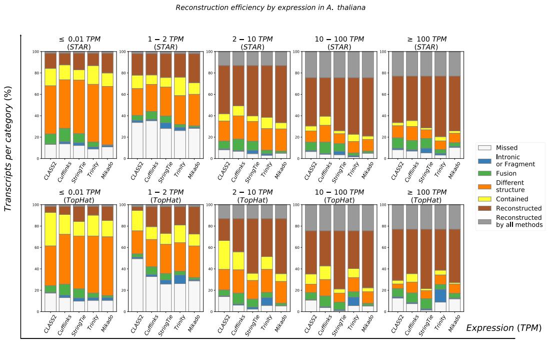

(a)

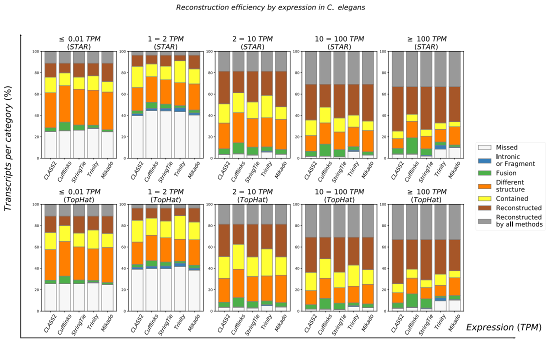

(b)

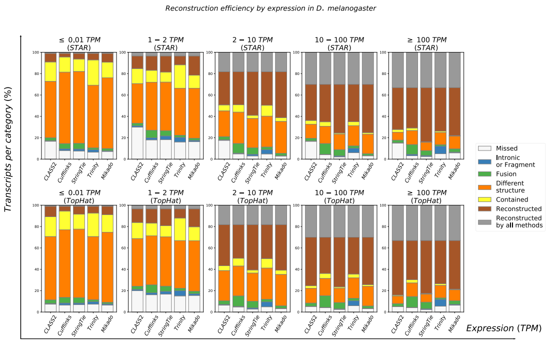

(c)

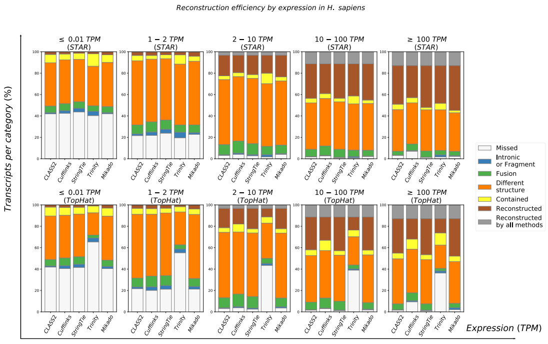

(d)

Figure SF5: Efficiency of reconstruction of the various assemblers on the basis of the transcript expression in *A. thaliana* (a), *C. elegans* (b), *D. melanogaster* (c) and *H. sapiens* (d).

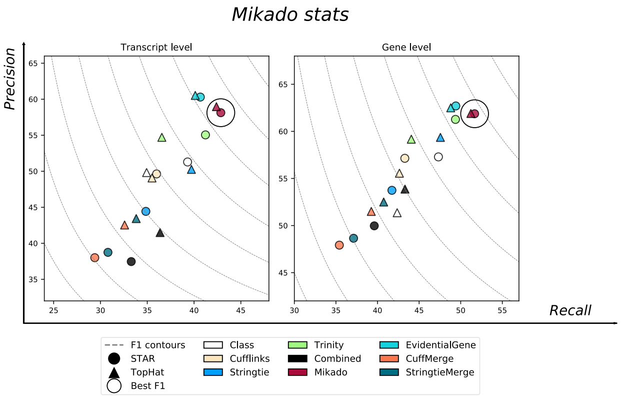

(a)

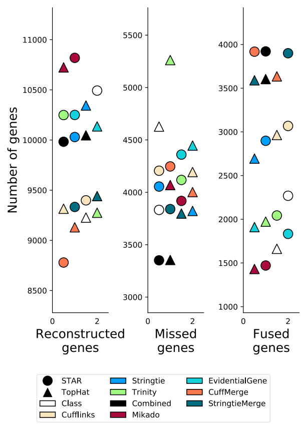

(b)

Figure SF6: a The increased accuracy of Mikado assemblies translates into a greater accuracy of the final annotation, as the polished evidence helps guiding the *ab initio* Augustus gene predictor. b MAKER is not capable of solving fusion genes present in the evidence, but rather, it will pass them through to the final annotation. By greatly reducing their number, Mikado is able to provide MAKER with evidence leading to less spurious chimeras.

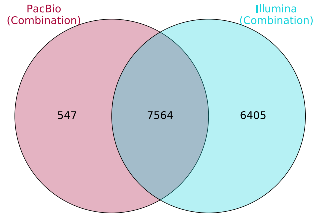

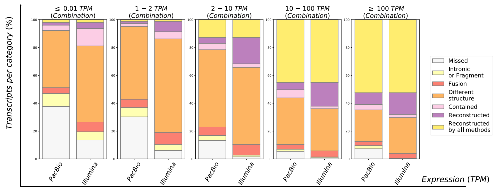

(b)

(a)

Figure SF7: a Venn diagram of genes reconstructed using Illumina or PacBio data. Although there is a significant overlap between the two technologies, both allow to reconstruct a sizable number of loci that would have been missed by excluding its alternative. b Illumina data is particularly effective at reconstructing genes with a mid to low expression level, whereas PacBio data is not comprehensive for this expression bracket. At higher expression values, PacBio data begins to display its increased accuracy when compared to Illumina.

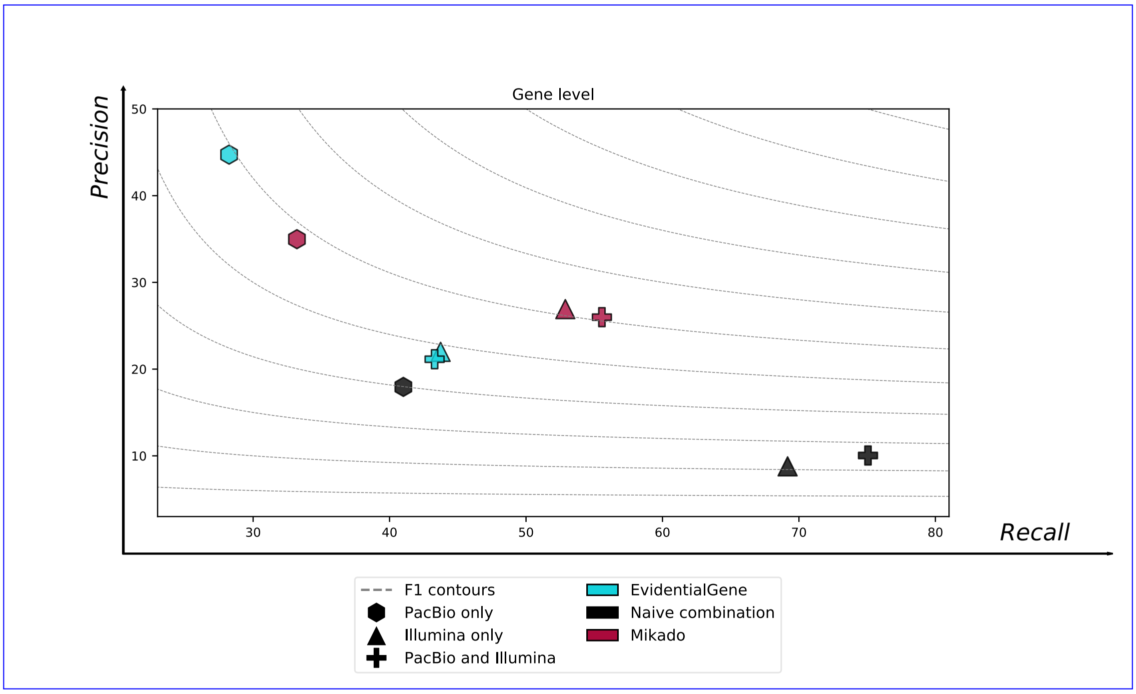

Figure SF8: **Analysis of mixtures of Illumina and PacBio reads.**

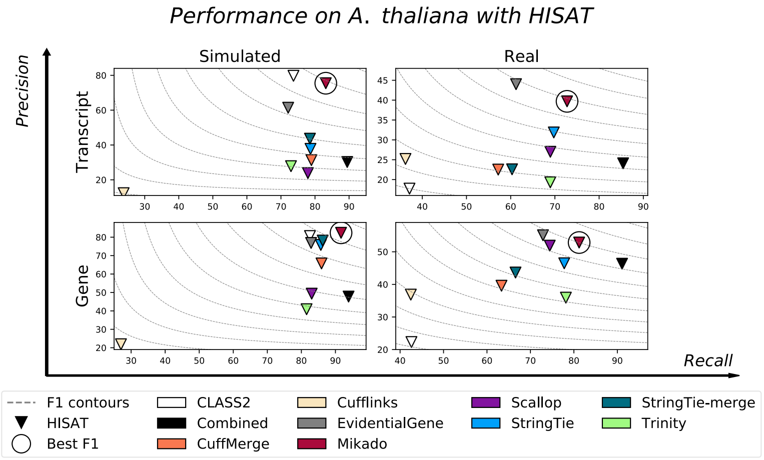

Figure SF9: **Performance of Mikado on real and simulated *A. thaliana* data, using HISAT2 as aligner and adding Scallop to the tested assemblers.**

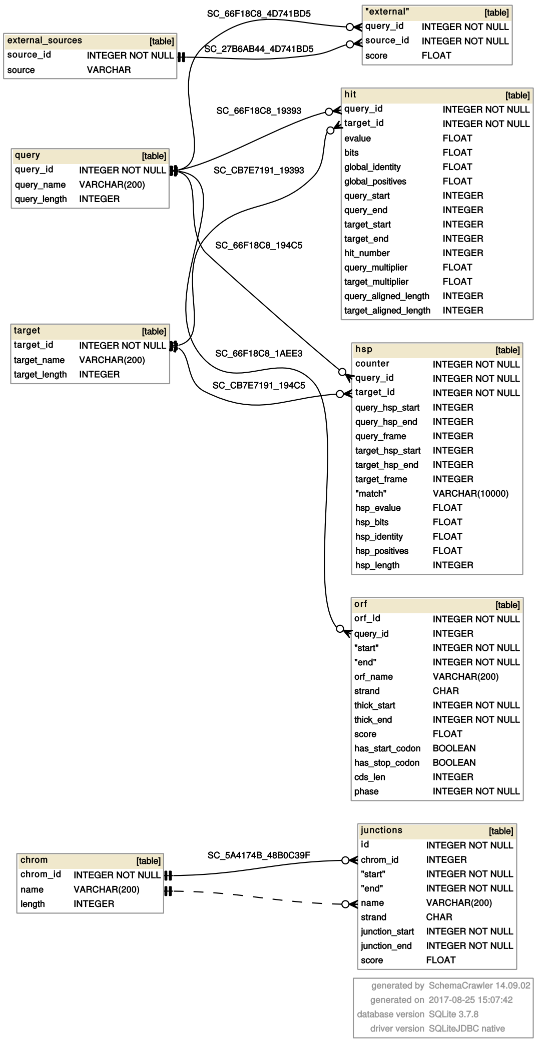

Figure SF10: **Schema of the database used internally by Mikado**

References

[1] Goodstein, D.M., Shu, S., Howson, R., Neupane, R., Hayes, R.D., Fazo, J., Mitros, T., Dirks, W., Hellsten, U., Putnam, N., Rokhsar, D.S.: Phytozome: a comparative platform for green plant genomics. Nucleic Acids Research **40**(D1), 1178–1186 (2012). doi:10.1093/nar/gkr944

[2] Phytozome: Phytozome archive (2017). `http://genome.jgi.doe.gov/pages/dynamicOrganismDownload.jsf?organism=Phytozome` Accessed 30/08/2017

[3] Hu, T.T., Pattyn, P., Bakker, E.G., Cao, J., Cheng, J.-F., Clark, R.M., Fahlgren, N., Fawcett, J.A., Grimwood, J., Gundlach, H., Haberer, G., Hollister, J.D., Ossowski, S., Ottilar, R.P., Salamov, A.A., Schneeberger, K., Spannagl, M., Wang, X., Yang, L., Nasrallah, M.E., Bergelson, J., Carrington, J.C., Gaut, B.S., Schmutz, J., Mayer, K.F.X., Van de Peer, Y., Grigoriev, I.V., Nordborg, M., Weigel, D., Guo, Y.-L.: The Arabidopsis lyrata genome sequence and the basis of rapid genome size change. Nature Genetics **43**(5), 476–481 (2011). doi:10.1038/ng.807

[4] DOE-JGI: Brassica rapa FPsc v1.3 (2017). http://phytozome.jgi.doe.gov/ Accessed 30/08/2017

[5] Slotte, T., Hazzouri, K.M., Ågren, J.A., Koenig, D., Maumus, F., Guo, Y.-L., Steige, K., Platts, A.E., Escobar, J.S., Newman, L.K., Wang, W., Mandáková, T., Vello, E., Smith, L.M., Henz, S.R., Steffen, J., Takuno, S., Brandvain, Y., Coop, G., Andolfatto, P., Hu, T.T., Blanchette, M., Clark, R.M., Quesneville, H., Nordborg, M., Gaut, B.S., Lysak, M.A., Jenkins, J., Grimwood, J., Chapman, J., Prochnik, S., Shu, S., Rokhsar, D., Schmutz, J., Weigel, D., Wright, S.I.: The Capsella rubella genome and the genomic consequences of rapid mating system evolution. Nature Genetics **45**(7), 831–835 (2013). doi:10.1038/ng.2669

[6] WormBase: WormBase WS251 (2015). ftp://ftp.wormbase.org/pub/wormbase/releases/WS251/species/ Accessed 2017-08-30

[7] EnsEMBL: EnsEMBL Metazoa v30 (2015). ftp://ftp.ensemblgenomes.org/pub/release-30/metazoa/ Accessed 2017-08-30

[8] EnsEMBL: EnsEMBL release 83 (2015). ftp://ftp.ensembl.org/pub/release-83/ Accessed 2017-08-30

[9] Khan, A., Mathelier, A.: Intervene: a tool for intersection and visualization of multiple gene or genomic region sets. BMC Bioinformatics **18**(1), 287 (2017). doi:10.1186/s12859-017-1708-7

[10] Lex, A., Gehlenborg, N., Strobelt, H., Vuillemot, R., Pfister, H.: Upset: visualization of intersecting sets. IEEE transactions on visualization and computer graphics **20**(12), 1983–1992 (2014)
