# Supplementary material for: Leveraging multiple transcriptome assembly methods for improved gene structure annotation
Source: Gigascience. 2018 Jul 24;7(8):giy093. doi: 10.1093/gigascience/giy093 (PMC6105091; doi:10.1093/gigascience/giy093)
Supplement: Response_to_Reviewer_Comments_Original_Submission.pdf [file giy093_response_to_reviewer_comments_original_submission.pdf]

Author's Response To Reviewer Comments

Close

Response to Editor

Dear Hans Zauner

We have now revised our manuscript addressing the comments of the reviewers. Including those points highlighted by yourself. We note that both reviewers indicated our tool addresses an important issue and thank them for their positive reviews, we have addressed the reviewers remaining queries and provide a point by point response in reply. We also describe additional data included in the revised manuscript, and provide a version with changes to the manuscript highlighted.

Editor comment 1. “One concern I'd like to highlight is the comment of both reviewers regarding the use of outdated tools, such as the assembler - please do consider to replace these with more up-to-date tools.”

To address the point relating to our choice of tools (used as an input to our approach and also to assess our method against) we have extended the Daijin pipeline to incorporate HISAT2 (aligner) and Scallop (assembler) and provide an additional supplementary figure (SF9) to show that our approach is amenable to new methods. However we note that the two reviewers queried different tools (reviewer 1 Tophat2, reviewer 2 cufflinks and cuffmerge) when making reference to potentially outdated tools; each of these tools continue to be cited even though new methods are available. Given Mikado is a way of integrating transcript assemblies, we feel it is important to show that our approach can incorporate data from both new and more established assemblers and that the approach is robust to data from methods that overall may not be individually the best. In total the paper evaluates our tool against nine alternative methods. We think this is substantive and believe removing popular tools that provide a familiar reference point would make it harder for gigascience readers to evaluate our method.

Editor comment 2 “Also the advice of reviewer 1 to package the tool with its dependencies e.g. via Bioconda seems a good suggestion to increase usability.”

Following the reviewer’s suggestion, Mikado and its companion tool Portcullis have been packaged into BioConda, from where they are now available. Mikado is present on the repository with the version used for the analyses in this article (v. 1.0.1) as well as with the updated versions 1.2. We plan to continue releasing future versions of the tool through this channel, as well as through GitHub and PyPI (from where Mikado was already available).

Editor comment 3. “In addition, please register any new software application in the SciCrunch.org database to receive a RRID (Research Resource Identification Initiative ID) number, and include this in your manuscript. This will facilitate tracking, reproducibility and re-use of your tool.”

Following the editor’s suggestion, Mikado has been registered on SciCrunch, and has been assigned the RRID SCR_016159. The “Availability of source code and requirements”

section has been updated to include both this RRID and the packaging in BioConda.

Reviewer reports:

Reviewer #1
Comment 1. “I can imagine that choosing the correct parameters is quite important to get high quality annotation output. However, this is not clearly described in the manuscript. For instance, how sensitive is the output to different scoring parameters? Will the ranking of mikado relative to other methods critically depend on parameter choice? From the documentation I gather that the scoring definition is quite flexible, which also makes it quite daunting. While I appreciate that careful species-specific optimization of the scoring falls outside the scope of this specific manuscript, but it would be good to at least discuss this.”

Mikado by design allows users to fine tune how transcripts will be selected to meet their own requirements, based on our experience of manually annotating genomes and assessing the output of various transcript assembly tools we have attempted to reproduce the choices manual annotators make in our selection of metrics and chosen scoring configuration. The pre-packaged scoring files for the four test species discussed in our manuscript only differ for a few metrics e.g. expected intron sizes, UTR length and proportion of UTR are increased in the human configuration to reflect the different characteristics of human genes. In addition, we increase the flank size for clustering transcripts into superloci for human compared to the more compact Arabidopsis, c.elegans and drosophila genomes. Running with the provided scoring files should represent a good starting point for most projects and we have added a section to the documentation explain how to configure the scoring (http://mikado.readthedocs.io/en/latest/Tutorial/Scoring_tutorial.html)and provide some example use cases (http://mikado.readthedocs.io/en/latest/Tutorial/Adapting.html). We now reference the documentation in the section of the manuscript summarising our method and provide a more detailed discussion in the conclusion about how Mikado can be used with different selection criteria and indicate clearly that these choices will influence the final transcript set.

Comment 2. “I strongly suggest that the authors make mikado (and all relevant dependencies) available through bioconda (see https://doi.org/10.1101/207092). While mikado itself is easy to install, I had a little more trouble with one of the dependencies (Portcullis).”

Mikado and its companion tool Portcullis have been packaged into BioConda, from where they are now available. Mikado is present on the repository with the version used for the analyses in this article (v. 1.0.1) as well as with the updated versions 1.1 and 1.2.x. We plan to continue releasing future versions of the tool through this channel, as well as through GitHub and PyPI (from where Mikado was already available)

Comment 3. “Is there a reason that Tophat is used instead of HISAT2? It is my understanding that HISAT2 has superseded TopHat2.”

We selected two of the most popular aligners available at the point we instigated the project, both STAR and Tophat2 continue to be well cited, with Tophat2 receiving ~1700 citations since 2017 (according to google scholar). We felt both aligners were familiar to potential users of our tool and had been utilised with a range of transcript assemblers by ourselves and

others in the literature. We acknowledge that HISAT2 and other more recently published aligners are available and have now incorporated HISAT2 as an option in our Daijin pipeline (that generates the required files for Mikado). We evaluated HISAT2, Tophat2 and STAR as part of assessing our portcullis tool https://doi.org/10.1101/217620 and while HISAT2 greatly reduced runtime compared to Tophat2, benefits were less clear cut in regard to recall and precision for spliced alignment (Fig 2 https://doi.org/10.1101/217620). As we anticipate new read aligners and assemblers will be developed, we have assessed mikado using assemblies generated from HISAT2 and include the recently published Scallop assembler doi:10.1038/nbt.4020. These assessments are included as supplementary figure SF9 and referred to in our conclusions to show that our approach it amenable to data from new methods and will continue to offer benefits over approaches using just a single assembler. We show Mikado to be effective with assemblies generated from HISAT2, STAR and Tophat2 so the choice of aligner is not crucial to our method.

Comment 4. "When describing the BLAST-assisted procedure, please also describe in the main text that you use proteins from related species in the benchmark. This is an important detail. I know it is clearly mentioned in the methods section, but I had to specifically look for it."

We have added the following sentence in the results, when explaining which procedure we followed to perform Mikado on the various datasets:

"For each of the four species under analysis, we also obtained reference-quality protein sequences from related species to inform the homology search through BLAST; details on our selection can be found in Table ST4."

Comment 5. "It would be helpful if all accession ids for the sequencing data were clearly mentioned under a header "data availability".

We now include the accession codes for the sequencing data under the section "Availability of supporting data and materials", with the following text:

"The sequencing runs analysed for this article can be found on ENA, under the accession codes PRJEB7093 (for A. thaliana) and PRJEB4208 (for the other other three species). The human sequencing data of our parallel Illumina and PacBio experiment can be found under the accession code PRJEB22606."

Comment 6. "p1: one of the most commonly used technology"

Now corrected

Reviewer #2

Comment 1. "Page 2, 1st column, lines 37-40. The authors indicate that Mikado will pick a representative transcript for each locus. It is unclear at this first mention, nor later in the manuscript (page 3, 2nd column, paragraph starting with line 43). I am under the impression that they do not exclude alternatively spliced isoforms that share exons, but the reference to excluding transcripts that overlap the primary transcript could be interpreted as such. Do they mean excluding transcripts that are entirely overlapping (and shorter) with the putative representative transcript? I think clearer language at first mention, and on page 3 would be

helpful. "

We agree with the reviewer that our description in the paper could have been clearer. We have amended the first paragraph to

"The software takes as input transcript structures in standard formats such as GTF and GFF3, with optionally BLAST similarity scores or a set of high quality splice junctions. Using this information, Mikado will then define gene loci and their associated transcripts. Each locus will be characterised by a primary transcript - ie the transcript in the region that best fits the requirements specified by the user. If any suitable alternative splicing event for the primary transcript is available, Mikado will add it to the locus. The software is written in python3 and Cython, and extensive documentation is available from https:/mikado.readthedocs.io/"

In the second paragraph, we have added the following sentences

"After the gene loci and associated primary transcripts have been defined, Mikado will look for potential alternative splicing events. Only transcripts that can be unambiguously assigned to a single gene locus will be considered for this phase. Mikado will add to the locus only transcripts whose structures are non-redundant with those already present, and which are valid alternative splicing events for the primary transcript, as defined by class codes (see http://mikado.readthedocs.io/en/latest/Usage/Compare.html#class-codes). Moreover, Mikado will discard any transcript whose score is too low when compared to the primary (by default, only transcripts with a score of 50% or more of the primary will be considered)."

"In the online documentation, we provide a discussion on how to customise scoring files according to the needs of the experimenter, and a tutorial to guide through its creation (http://mikado.readthedocs.io/en/latest/Tutorial/Adapting.html)."

We hope that this additional text will make the context clearer to the reader.

Comment 2. "A substantial issue I have is that the authors employ outdated assemblers in their analyses. Cufflinks is notably poor at reconstructing transcripts, and has been replaced by StringTie, both of which were developed by the same group. I would prefer if Cufflinks were excluded entirely, and/or replaced with another transcript assembly tool."

We selected four assembly tools for use in our study, in addition to providing a reference point to evaluate Mikado they are also the input to our tool. We thought it important to show that Mikado was robust to incorporating assemblies from a range of sources and qualities, as such we selected two more recently published assemblers in Stringtie and CLASS that we had used in our own studies and which we believed to perform well (this is now borne out by the results described in our manuscript), in addition we also selected what we considered to be the most popular de novo and reference guided transcript assemblers in Trinity and Cufflinks (Cufflinks has 388 citations this year and nearly 7000 overall). We agree with the reviewer that Cufflinks is inferior to Stringtie and therefore would not be our choice if selecting a single method, however, one of the key arguments for our approach is that even though a method may overall be inferior it may still reconstruct transcripts missed by other methods. We have now include set analysis of fully reconstructed transcripts showing the intersections between the assembly methods (figure SF4), for each of the four species and

two aligners tested, cufflinks identifies between 218 and 1075 transcript not reconstructed fully by any other method, for H.sapiens with Tophat2 alignments Cufflinks generated the highest number of fully-reconstructed transcripts specific to a single method (1058). We believe this merits including cufflinks in our analysis, additionally as a tool still widely used and familiar to gigascience readers we believe it provides a useful reference point. As new methods will continue to be developed we have assessed mikado using assemblies generated from HISAT2 and include the recently published Scallop assembler doi:10.1038/nbt.4020. These assessments are included as supplementary figure SF9 and referred to in our conclusions to show that our approach it amenable to data from new methods and will continue to offer benefits over approaches using just a single assembler.

Added figure SF4 (Upset plots) and the following sentence in the results:

"Closer inspection of the data shows, this effect is not due to a single assembler having greater efficiency; rather, each of the tools is shown to be the only one capable of correctly reconstructing hundreds of the expressed transcripts (Supplementary Figure 4)."

Comment 3. "Page 2, line 13, 2nd column. It should be made clear that the number of "reconstructed transcripts" both in the text and in the legend of figure SF1 is the number of predicted transcripts, not the underlying, true reference transcripts. It becomes clearer a while later when discussing recall and precision, and it should be clear given that more transcripts are predicted for fly than exist in the actual annotation, but, well, best to be as clear as possible!"

We have edited to make this clearer in the text and figure legend

"The number of transcripts assembled varied substantially across methods, with StringTie and Trinity generally reporting a greater number of transcripts (Supplementary Figure SF1)"

Figure SF1: Number of genes and transcripts assembled for each method and species.

Comment 4. "Page 2, 2nd column, line 33. How are "erroneous transcripts" defined? Are they simply real but missing from the genome assembly, or low coverage transcriptional noise? Speaking from experience, I have used de novo transcriptome assemblies to produce valid CDS with strong matches to Uniref peptides that correspond to annotated genes in closely related species."

We have updated the text to make this clearer

"In contrast, Trinity and StringTie often outperformed the recall of CLASS2, but were also much more prone to yield transcripts absent from the curated public annotations (Supplementary Figure SF2, SF3). Although many of these might be real, yet-unknown transcripts, the high number of chimeric transcripts suggests to treat these novel models with suspicion."

More generally we agree with the reviewer that the reference annotations will be incomplete and therefore determining true precision based on real data is challenging. We chose species with curated and regularly updated gene sets to provide the best reference annotation for our

assessment. In addition the results presented later in the manuscript using simulated reads (where precision can be determined precisely) supports the statement regarding higher precision for CLASS2 over Trinity and Stringtie.

Comment 5. “Is there any flexibility in the nature of the input data provided to Mikado, i.e. could one use splice junctions defined by tools other than Portcullis, etc. Or is the Snakemake pipeline not so flexible in this regard? I ask because new tools are being developed all the time, and allowing to replace tools that Mikado wraps will allow it stay current well into the future.”

We agree with the reviewer that the field is very dynamic, with the popularity and usefulness of tools changing very quickly. To prevent obsolescence, Mikado was written so that it can accept input from any assembler as long as it follows the very widespread and standard GTF, GFF3 or BED12 file formats. The “prepare” step of the pipeline will then uniform the data contained in these files. With regard to the set of reliable splice junctions, these can be generated by any method and provided in BED12 format. The Mikado part of the Daijin pipeline should therefore be robust to development in the field; as long as the input files are in a supported format Mikado will be able to use them. This flexibility allows Mikado to be applied in different contexts; although it is outside of the scope of this article, in a different project we applied Mikado to merge and select from two ab initio prediction datasets. This was possible thanks to the agnostic nature of the first step. The first part of the Daijin pipeline, which is tasked with aligning and assembling reads i.e. generating the input for Mikado, includes prescribed options for both alignment and assembly. However, new tools can be added to the workflow quite easily - as is typical with the SnakeMake pipelines, adding a rule will in most cases suffice. The code is public, and we plan to add other tools in time; for example, we recently added Scallop.

Comment 6. “In the Performance of Mikado section, there is a reference to the low coverage filter for CLASS2 that allows it perform better in a particular case. In the next section, the authors refer to Mikado's ability to do such filtering. In the Performance section, they should edit to say something to the effect of , "Mikado can be implemented using coverage and other filters to improve assembly performance (as described in the next section)."

The sections the reviewer is refering to do not address the same issue. The reference to CLASS2 is in relation to it’s higher precision by virtue of not assembling models from intronic sequences that were incorrectly assembled by other methods. The next section on filtering lenient assemblies relates specifically to generating transcript assemblies with a lower minimum isoform fraction and using Mikado to filter these. It would be possible to configure MIkado to reduce the number of intronic fragments based on filtering for low coverage but Mikado does not “out of the box” use coverage to score or select transcripts. We do enable the user to provide external metrics (http://mikado.readthedocs.io/en/latest/Algorithms.html?highlight=external-metrics#external-metrics) that could then be used to score coverage/expression and used in conjunction with how Mikado identifies and excludes what we refer to as fragments (section 8.1http://mikado.readthedocs.io/en/latest/Algorithms.html#picking-transcripts-how-to-define-loci-and-their-members). We have updated the text to provide reference to this.

“While Mikado does not calculate or utilise coverage to score and select transcripts, we do make provision for externally generated metrics that could be used in conjunction with Mikado’s fragment filtering to screen out lowly expressed intronic models.”

Comment 7. “Also in the Performance section, similar to my complaint about Cufflinks above, CuffMerge is an outdated tool.”

We disagree with the reviewer that cuffmerge is an outdated tool as part of the same suite as cufflinks this set of tools continue to be well cited (see earlier reply). In the section describing multi sample transcript reconstruction we assessed Mikado against four alternative methods of which cuffmerge is one. We selected a recently published tool in TACO developed specifically for this purpose, established tools in StringtieMerge and CuffMerge, and based on our own positive experience EvidentialGene. While not the best performing tool in any context, cuffmerge did generate higher F1 scores than alternative methods on some of the datasets used. We believe this merits its inclusion and that the four methods provide a good reference point to assess our own tool against. We do not see that the manuscript would be improved by excluding the cuffmerge results.

Comment 8. “Page 5, column 2, ~ line 36, reference to Figure SF8 should be to Figure SF7”.

The reviewer was indeed correct in pointing this out. However, since we added a new Supplementary Figure (SF4), the numbering of following figures was changed as well. We have checked and revised as needed the numbering throughout the manuscript. In this specific case, the new correct numbering is indeed SF8.

Comment 9. “Is there a plan for further development (and debugging if necessary) of Mikado, and user support (e.g. via a Google discussion group, etc.)? Too many potentially valuable bioinformatics projects have an effective lifespan of the doctoral or postdoctoral programs of the primary developers, with empty wiki pages that say "coming soon" that haven't been updated in years. “

The reviewer is indeed correct in pointing out that too many tools in the bioinformatics are developed and then left to languish Regarding the current levels of user support and development, we can point to our levels of support through the “Issues” page on the GitHub project (https://github.com/lucventurini/mikado/issues?utf8=%E2%9C%93&q=is%3Aissue+), which is our main point of collection for bug reports and feature requests. We try to be active in replying to our user-base and correct bugs as quickly as possible. Software development is also active; we released two major releases in the past year, and do not plan to end development any time soon. In more general terms, Mikado has been created with the purpose of being central in the annotation pipelines developed at our institute, not just as a postdoctoral project. As such, our institute is committed to continue the maintenance and the development of the software tool irrespective of the professional fate of the manuscript’s authors. For this reason, we strived to document extensively the code base, follow formally correct software writing style, and include extensive unit and system-test coverage. These provisions, and the nature of Mikado as an important key for future annotation pipelines of the institute, should make the tool much more robust than most.

Comment 10. “With respect to parameter tuning for Mikado Pick described on page 8, it would be useful to provide some guidelines re: deviating from the default scoring scheme, perhaps by a short paragraph on the matter, and then an expanded discussion on gh the github page. With more and more groups assembling genomes and needing to annotate

them, the researchers launching annotation analyses probably will not have the depth of bioinformatics experience to do a good job of changing scoring schemes, leading to use of default settings that may lead to sub-optimal settings. A simple tutorial/walkthrough page on the github repo would get such groups on the right track.”

The pre-packaged scoring files for the four test species discussed in our manuscript only differ for a few metrics e.g. expected intron sizes, UTR length and proportion of UTR are increased in the human configuration to reflect the different characteristics of human genes. In addition, we increase the flank size for clustering transcripts into superloci for human compared to the more compact arabidopsis, c.elegans and drosophila genomes. Running with the provided scoring files should represent a good starting point for most projects and we have added a section to the documentation explain how to configure the scoring file (http://mikado.readthedocs.io/en/latest/Tutorial/Scoring_tutorial.html) and giving some example use cases (https://mikado.readthedocs.io/en/latest/Tutorial/Adapting.html).

Close
