# Supplementary material for: Leveraging multiple transcriptome assembly methods for improved gene structure annotation
Source: Gigascience. 2018 Jul 24;7(8):giy093. doi: 10.1093/gigascience/giy093 (PMC6105091; doi:10.1093/gigascience/giy093)
Supplement: Response_to_Reviewer_Comments_Revision_1.pdf [file giy093_response_to_reviewer_comments_revision_1.pdf]

Author's Response To Reviewer Comments

Close

We have incorporated all changes described in your e-mail below

Dear David,

The supporting GigaDB dataset for your manuscript "Leveraging multiple transcriptome assembly methods for improved gene structure annotation" (GIGA-D-18-00017R1) is now released:

http://gigadb.org/dataset/view/id/100464/File_page/2

and we can now proceed to formally accept and publish your manuscript.
Before acceptance, please include these minor revisions in your manuscript:

1) Please add the citation of the the GigaDB dataset to your bibliography:

[xx] Venturini L; Caim S; Kaithakottil GG; Mapleson DL; Swarbreck D. Supporting data for "Leveraging multiple transcriptome assembly methods for improved gene structure annotation" GigaScience Database 2018. http://dx.doi.org/10.5524/100464

Please cite your dataset from the "data availability" section of the manuscript and elsewhere, if appropriate.

2) Please also turn other URLs mentioned in your text (github, figshare) into citations - that is, please give each URL a numbered reference in the bibliography and cite it from the main text.

3) I see you got a RRID for your tool, that's great - please mention this in the "availability of source code" section of the manuscript.

Please also ensure that your revised manuscript conforms to the journal style, which can be found in the Instructions for Authors on the journal homepage.

Once you have made the necessary corrections, please submit a revised manuscript online at:

https://giga.editorialmanager.com/

If you have forgotten your username or password please use the "Send Login Details" link to get your login information. For security reasons, your password will be reset.

We look forward to publishing your revised manuscript.

Best wishes,

Hans

Hans Zauner
GigaScience
www.gigasciencejournal.com

Close
