# Supplementary material for: Leveraging multiple transcriptome assembly methods for improved gene structure annotation
Source: Gigascience. 2018 Jul 24;7(8):giy093. doi: 10.1093/gigascience/giy093 (PMC6105091; doi:10.1093/gigascience/giy093)
Supplement: Reviewer_1_Report_(Original_Submission) -- Simon van Heeringen, Ph.D. — 13 Feb 2018 Reviewed [file giy093_reviewer_1_report_(original_submission).pdf]

Reviewer Report

Title: **Leveraging multiple transcriptome assembly methods for improved gene structure annotation**

Version: **Original Submission** **Date:** 2/13/2018

Reviewer name: **Simon van Heeringen, Ph.D.**

Reviewer Comments to Author:

The manuscript "Leveraging multiple transcriptome assembly methods for improved structure annotation" describes mikado, a method to improve gene annotation by an ensemble approach.With the improvements in genome sequencing and assembly technologies, it is becoming much more feasible to obtain a genome assembly for a species of interest. This makes gene annotation a relevant problem. Mikado addresses this by combining the output of existing RNA-seq transcript reconstruction methods to improve the resulting predicted gene structure. The manuscript addresses an important issue and is well-written. The analyses are clear, detailed and informative. The mikado source code is available and looks to be in excellent state. There is exhaustive documentation and a high fraction of the code is covered by tests. In my opinion both the software and the manuscript are of high quality. With the documentation I could run the software on my own data without any major hurdles. I have some comments which are listed below.Major comments1. I can imagine that choosing the correct parameters is quite important to get high quality annotation output. However, this is not clearly described in the manuscript. For instance, how sensitive is the output to different scoring parameters? Will the ranking of mikado relative to other methods critically depend on parameter choice? From the documentation I gather that the scoring definition is quite flexible, which also makes it quite daunting. While I appreciate that careful species-specific optimization of the scoring falls outside the scope of this specific manuscript, but it would be good to at least discuss this.2. I strongly suggest that the authors make mikado (and all relevant dependencies) available through bioconda (see https://doi.org/10.1101/207092). While mikado itself is easy to install, I had a little more trouble with one of the dependencies (Portcullis).Minor comments1. Is there a reason that Tophat is used instead of HISAT2? It is my understanding that HISAT2 has superseded Tophat.2. When describing the BLAST-assisted procedure, please also describe in the main text that you use proteins from related species in the benchmark. This is an important detail. I know it is clearly mentioned in the methods section, but I had to specifically look for it.3. It would be helpful if all accession ids for the sequencing data were clearly mentioned under a header "data availability".4. p1: "one of the most commonly used technology"

Level of Interest

Please indicate how interesting you found the manuscript: An article of importance in its field

Quality of Written English

Please indicate the quality of language in the manuscript: Acceptable

Declaration of Competing Interests

Please complete a declaration of competing interests, considering the following questions:

- Have you in the past five years received reimbursements, fees, funding, or salary from an organisation that may in any way gain or lose financially from the publication of this manuscript, either now or in the future?
- Do you hold any stocks or shares in an organisation that may in any way gain or lose financially from the publication of this manuscript, either now or in the future?
- Do you hold or are you currently applying for any patents relating to the content of the manuscript?
- Have you received reimbursements, fees, funding, or salary from an organization that holds or has applied for patents relating to the content of the manuscript?
- Do you have any other financial competing interests?
- Do you have any non-financial competing interests in relation to this paper?

If you can answer no to all of the above, write 'I declare that I have no competing interests' below. If your reply is yes to any, please give details below.

I declare that I have no competing interests

I agree to the open peer review policy of the journal. I understand that my name will be included on my report to the authors and, if the manuscript is accepted for publication, my named report including any attachments I upload will be posted on the website along with the authors' responses. I agree for my report to be made available under an Open Access Creative Commons CC-BY license (http://creativecommons.org/licenses/by/4.0/). I understand that any comments which I do not wish to be included in my named report can be included as confidential comments to the editors, which will not be published.

I agree to the open peer review policy of the journal

To further support our reviewers, we have joined with Publons, where you can gain additional credit to further highlight your hard work (see: https://publons.com/journal/530/gigascience). On publication of this paper, your review will be automatically added to Publons, you can then choose whether or not to claim your Publons credit. I understand this statement. yes
