# Supplementary material for: Leveraging multiple transcriptome assembly methods for improved gene structure annotation
Source: Gigascience. 2018 Jul 24;7(8):giy093. doi: 10.1093/gigascience/giy093 (PMC6105091; doi:10.1093/gigascience/giy093)
Supplement: Reviewer_2_Report_(Original_Submission) -- Adam Freedman, Ph.D. — 2/28/2018 Reviewed [file giy093_reviewer_2_report_(original_submission).pdf]

Reviewer Report

Title: **Leveraging multiple transcriptome assembly methods for improved gene structure annotation**

Version: **Original Submission** **Date:** 2/28/2018

Reviewer name: **Adam Freedman, Ph.D.**

Reviewer Comments to Author:

The authors present a new method for integrating evidence from different transcript assembly methods to leverage the complementarity of different assemblies, capitalizing on their individual strengths and minimizing the effects of their individual weaknesses. While I have not yet tested Mikado myself, the results the authors present suggest the tool has much promise for improving the outputs of annotation pipelines. I have no major methodological issues with Mikado, as presented, but have a number of other issues I would like the authors to address. They are listed below, mostly in order as they appear in the manuscript.1. Page 2, 1st column, lines 37-40. The authors indicate that Mikado will pick a representative transcript for each locus. It is unclear at this first mention, nor later in the manuscript (page 3, 2nd column, paragraph starting with line 43). I am under the impression that they do not exclude alternatively spliced isoforms that share exons, but the reference to excluding transcripts that overlap the primary transcript could be interpreted as such. Do they mean excluding transcripts that are entirely overlapping (and shorter) with the putative representative transcript? I think clearer language at first mention, and on page 3 would be helpful. 2. A substantial issue I have is that the authors employ outdated assemblers in their analyses. Cufflinks is notably poor at reconstructing transcripts, and has been replaced by StringTie, both of which were developed by the same group. I would prefer if Cufflinks were excluded entirely, and/or replaced with another transcript assembly tool. 3. Page 2, line 13, 2nd column. It should be made clear that the number of "reconstructed transcripts" both in the text and in the legend of figure SF1 is the number of predicted transcripts, not the underlying, true reference transcripts. It becomes clearer a while later when discussing recall and precision, and it should be clear given that more transcripts are predicted for fly than exist in the actual annotation, but, well, best to be as clear as possible!4. Page 2, 2nd column, line 33. How are "erroneous transcripts" defined? Are they simply real but missing from the genome assembly, or low coverage transcriptional noise? Speaking from experience, I have used de novo transcriptome assemblies to produce valid CDS with strong matches to Uniref peptides that correspond to annotated genes in closely related species. 5. Is there any flexibility in the nature of the input data provided to Mikado, i.e. could one use splice junctions defined by tools other than Portcullis, etc. Or is the Snakemake pipeline not so flexible in this regard? I ask because new tools are being developed all the time, and allowing to replace tools that Mikado wraps will allow it stay current well into the future. 6. In the Performance of Mikado section, there is a reference to the low coverage filter for CLASS2 that allows it perform better in a particular case. In the next section, the authors refer to Mikado's ability to do such filtering. In the Performance section, they should edit to say something to the effect of , "Mikado can be implemented using coverage and other filters to improve assembly performance (as described in the next section)."7. Also in the Performance section, similar to my complaint about Cufflinks above, CuffMerge is an outdated tool. 8. Page 5, column 2, ~ line 36, reference to Figure SF8 should be to Figure SF7.9. Is there a plan for further development (and debugging if necessary) of Mikado, and user support (e.g. via a Google discussion group, etc.)? Too many potentially valuable bioinformatics projects have an effective lifespan of the doctoral or postdoctoral programs of the primary developers, with empty wiki pages that say "coming soon" that haven't been updated in years. 10. With respect to parameter tuning for Mikado Pick described on page 8, it would be useful to provide some guidelines re: deviating from the default scoring scheme, perhaps by a short paragraph on the matter, and then an expanded discussion on gh the github page. With more and more groups assembling genomes and needing to annotate them, the researchers launching annotation analyses probably will not have the depth of bioinformatics experience to do a good

job of changing scoring schemes, leading to use of default settings that may lead to sub-optimal settings. A simple tutorial/walkthrough page on the github repo would get such groups on the right track.

Level of Interest

Please indicate how interesting you found the manuscript: An article of importance in its field

Quality of Written English

Please indicate the quality of language in the manuscript: Acceptable

Declaration of Competing Interests

Please complete a declaration of competing interests, considering the following questions:

- Have you in the past five years received reimbursements, fees, funding, or salary from an organisation that may in any way gain or lose financially from the publication of this manuscript, either now or in the future?
- Do you hold any stocks or shares in an organisation that may in any way gain or lose financially from the publication of this manuscript, either now or in the future?
- Do you hold or are you currently applying for any patents relating to the content of the manuscript?
- Have you received reimbursements, fees, funding, or salary from an organization that holds or has applied for patents relating to the content of the manuscript?
- Do you have any other financial competing interests?
- Do you have any non-financial competing interests in relation to this paper?

If you can answer no to all of the above, write 'I declare that I have no competing interests' below. If your reply is yes to any, please give details below.

I declare that I have no competing interests.

I agree to the open peer review policy of the journal. I understand that my name will be included on my report to the authors and, if the manuscript is accepted for publication, my named report including any attachments I upload will be posted on the website along with the authors' responses. I agree for my report to be made available under an Open Access Creative Commons CC-BY license (http://creativecommons.org/licenses/by/4.0/). I understand that any comments which I do not wish to be included in my named report can be included as confidential comments to the editors, which will not be published.

I agree to the open peer review policy of the journal

To further support our reviewers, we have joined with Publons, where you can gain additional credit to further highlight your hard work (see: https://publons.com/journal/530/gigascience). On publication of this paper, your review will be automatically added to Publons, you can then choose whether or not to claim your Publons credit. I understand this statement. yes
